# Autophagy Induced by Palmitic Acid Regulates Neutrophil Adhesion Through the Granule-Dependent Degradation of αMβ2 Integrin in Dairy Cows With Fatty Liver

**DOI:** 10.3389/fimmu.2021.726829

**Published:** 2021-10-07

**Authors:** Zhicheng Peng, Chenxu Zhao, Xiliang Du, Yuchen Yang, Yunfei Li, Yuxiang Song, Baochen Fang, Yuming Zhang, Xia Qin, Yuanyuan Zhang, Xiaobing Li, Zhe Wang, Xinwei Li, Guowen Liu

**Affiliations:** ^1^ Key Laboratory of Zoonosis, Ministry of Education, College of Veterinary Medicine, Jilin University, Changchun, China; ^2^ College of Food Science and Engineering, Jilin University, Changchun, China

**Keywords:** fatty liver dairy cow, palmitic acid, neutrophil, granule, autophagy, vacuolation, αMβ2 integrin, adhesion

## Abstract

β2 integrins are critical for neutrophil firm adhesion, trans-endothelial migration, and the recruitment to the inflamed tissue. Autophagy is implicated in cell migration and tumor metastasis through facilitating the turnover of β1 integrins; however, whether autophagy is able to control neutrophil migration by promoting the degradation of β2 integrins is unexplored. Here, we show that high blood levels of palmitic acid (PA) strongly triggered neutrophil autophagy activation, leading to adhesion deficiency in dairy cows with fatty liver. The three neutrophil granule subtypes, namely, azurophil granules (AGs), specific granules (SGs), and gelatinase granules (GGs), were engulfed by the autophagosomes for degradation, resulting in an increased vacuolation in fatty liver dairy cow neutrophils. Importantly, the adhesion-associated molecules CD11b and CD18 distributed on AGs, SGs, and GGs were degraded with the three granule subtypes by autophagy. Moreover, FGA, Hsc70, and TRIM21 mediated the degradation of cytosolic oxidized–ubiquitinated CD11b and CD18. Collectively, our results demonstrate that high blood PA triggers neutrophil autophagy-dependent vacuolation and granule-dependent adhesion deficiency, decreasing neutrophil mobility, and impairing the innate immune system of dairy cow with fatty liver. This theory extends the category of autophagy in maintaining granule homeostasis and provides a novel strategy to improve the immune of dairy cows with metabolic disease.

## Introduction

Neutrophils are highly mobile, and the most abundant innate immune cells. Mature neutrophils are densely packed with three granule subunits, including azurophil granules (AGs), specific granules (SGs), and gelatinase granules (GGs), each of which is an armory of adhesion-related molecules, antimicrobials, and various hydrolytic enzymes (1). As known, AGs, SGs, and GGs can be distinguished by their marker proteins myeloperoxidase (MPO), lactoferrin and matrix metalloprotease-9 (MMP-9), respectively (2–5). Physiologically, neutrophils roll and adhere along the vessel walls to conduct immune surveillance. Once activated, the circulating neutrophils undergo extravasation from the bloodstream into the inflamed sites to protect against invading microorganisms.

Many β2 integrins are expressed on neutrophils, including CD11a/CD18 (αLβ2), CD11b/CD18 (αMβ2), CD11c/CD18 (αXβ2), and CD11d/CD18 (αDβ2), which mediate neutrophil firm adhesion, trans-endothelial migration and the recruitment to inflamed sites (6–8). CD11b and CD18 are the most abundant and critical β2 integrins of dairy cows during these processes (9, 10). The reduction of the CD11b and CD18 on neutrophils due to excessive degradation or genetic damage leads to leukocyte adhesion deficiency (LAD), which inevitably causes immunodeficiency, as shown by recurrent bacterial infections and extending wound healing, such as mastitis and endometritis (11–13).

Autophagy is a lysosome-dependent degradation process by which the cytosolic oxidized–ubiquitinated proteins and various damaged organelles (mitochondria) are encapsulated and delivered to lysosomes for degradation (14). It has been suggested that neutrophil vacuolation might be associated with autophagy-triggered granule fusion events (15). Additionally, inflammation-induced proteins, lysosome-associated membrane proteins (LAMPs), and the autophagic receptor p62 have been reported to be observed on the granules (1). Furthermore, autophagy deficiency reduces the neutrophil-mediated inflammation and degranulation (16). Taken together, these results lead us to hypothesize that autophagy may be associated with the degradation of the AGs, SGs, and GGs. Coincidentally, CD11b and CD18 were also observed on the three granule subunits, and these molecules might be degraded together with these granules, though the specific mechanism is unknown (1).

Fatty liver in dairy cows is a highly prevalent condition that affects above 50% of the lactation dairy cows with manifestations and complications in over-inflammation and innate immunosuppression, including access neutrophil recruitment impairment (17, 18). The cows with fatty liver demonstrate innate immune suppression that is characterized by a higher susceptibility for these cows to infectious diseases, such as mastitis and endometritis (17). Increased vacuolated neutrophils were observed in ethanol toxified and septicemia patients due to the changes in their blood constituents, which might be associated with autophagy-dependent degradation of granules (19, 20). The neutrophil granules play a crucial role in clearing invading microbes and neutrophil adhesion, as antimicrobials and adhesion-related proteins are stored in them (21). So, an increased vacuolated neutrophil is an indicator of an inflamed status, because of exhaustion of granular compartments. High blood levels of non-esterified fatty acids (NEFAs) are the main pathological characteristics of dairy cows with fatty liver. PA is a significant component of NEFAs, which is a saturated fatty acid with high lipotoxicity, which strongly induces autophagy. However, how high blood PA triggers fatty liver dairy cow neutrophil autophagy-dependent vacuolation and adhesion deficiency and impairs immune function is unexplored.

Here, we identified that high blood PA triggers neutrophil autophagy activation and thus leads to granules being delivered to the lysosomes for degradation, leading to an increased neutrophil vacuolation in fatty liver dairy cows. Notably, the adhesion-associated molecules CD11b and CD18 distributed on the AGs, SGs, and GGs are degraded together with the three granule subunits, eventually inducing neutrophil adhesion and diapedesis deficiency, *ex vivo* and *in vitro*. Moreover, integrin ligand protein, FGA, molecular chaperone protein, Hsc70, and TRIM21 mediate the turnover of a fraction of cytoplasmic oxidized–ubiquitinated CD11b and CD18. These findings demonstrate autophagy as an important regulator of neutrophil mobility and provide a theoretical basis to elucidate the innate immunosuppression of the dairy cows with fatty liver.

## Materials and Methods

### Animals and Samples

Histopathologic diagnoses for fatty liver dairy cows were undertaken in accordance with characteristic histological features. Health (*n* = 12) and fatty liver (*n* = 12) Holstein dairy cows were selected from a 1000-cow dairy farm located in Suihua City, Heilongjiang Province, China. Routine physical examination, including a determination of the rectal temperature, respiratory rate, and pulse rate were performed on each cow, to exclude the cows with other comorbidities. Anesthetics were used to ensure animal welfare. Normal and fatty liver cow venous blood and liver tissue samples were collected and then the pathological diagnosis was performed. Twelve normal and 12 fatty liver dairy cows were selected randomly from the normal and fatty liver dairy cows. The clinical parameters of health and fatty liver cows are listed as (**Table 1**). All protocols were performed in accordance with guidelines of the Ethics Committee on the Use and Care of Animals at Jilin University (Changchun, China).

**Table 1:** The biochemical parameters and clinical characteristics of normal and fatty liver dairy cows.

Parameter	Normal cows (*n* = 12)	Fatty liver cows (*n* = 12)	*p*-value
BW (kg)	684 ± 11	682 ± 11	0.910
BCS	2.91 ± 0.075	3.09 ± 0.085	0.127
Milk production (kg of milk/cow per day)	30.59 ± 0.56	29.81 ± 0.59	0.342
DMI (kg/day)	19.9 ± 0.30	20.2 ± 0.33	0.523
Fat	3.55 ± 0.045	3.68 ± 0.064	0.093
Protein	3.12 ± 0.058	3 ± 0.068	0.214
Lactose	4.8 ± 0.037	4.62 ± 0.049**	0.008
NEFAs (mM)	0.25 ± 0.034	1.08 ± 0.11**	0.000
PA (mM)	0.1 ± 0.014	0.36 ± 0.032**	0.000
BHBA (mM)	0.74 ± 0.11	1.43 ± 0.22**	0.009
GLU (mM)	3.22 ± 0.10	2.52 ± 0.20**	0.005
AST (U/L)	73 ± 6.25	110.75 ± 6.72**	0.000
ALT (U/L)	33.08 ± 2.35	19.33 ± 1.51**	0.000
AST/ALT	2.41 ± 0.35	5.95 ± 0.44**	0.000
HP (g/L)	0.17 ± 0.017	0.49 ± 0.029**	0.000
SAA (μg/ml)	46.53 ± 2.77	80.56 ± 4.16**	0.000
GLDH (U/L)	19.6 ± 1.02	41.83 ± 2.16**	0.000
GGT (U/L)	12.34 ± 0.40	23.39 ± 1.99**	0.000
White blood cells (10^9^/L)	13.16 ± 1.46	11.77 ± 1.26	0.478
Neutrophils (10^9^/L)	9.19 ± 1.16	7.9 ± 1.08	0.425
Neutrophils/White blood cells (%)	70.18 ± 3.41	65.59 ± 2.64	0.300
Lymphocytes (10^9^/L)	1.92 ± 0.16	2.28 ± 0.20	0.177
Monocytes(10^9^/L)	0.19 ± 0.074	0.18 ± 0.018	0.829

(**p < 0.01 versus normal dairy cows).

### Dairy Cow Peripheral Blood Neutrophil Isolation

Bovine peripheral neutrophils were isolated using commercialized kit purchased from TBDsciences (Tianjin, China) according to the manufacturer’s protocol. The viability (>98%) was assessed by trypan blue staining. Cell purity (>95%) was analyzed by Wright and Giemsa staining and flow cytometry based on CD11b expression.

### HL-60, Antibodies, and Reagents

The HL-60 cell line and Calcein AM (KGAF001) were purchased from Keygentec company. Bovine peripheral blood neutrophil isolation kits (LZS1094) were purchased from TBDsciences. RPMI-1640 medium was purchased from Gibco. Anti-Mouse β2/CD18 (Ab657), Anti-Mouse β2/CD18 (Ab3917), Anti-Mouse Hsc70 (Ab2788), Anti-rabbit Fibrinogen alpha chain (ab92572), Anti-Rabbit LC3A/B (Ab128025), Anti-Rabbit SQSTM1/p62 (Ab101266), Anti-Rabbit MMP-9 (PA5-27191), and Anti-goat Lactoferrin (Ab112968) were purchased from Abcam. Anti-Rabbit MPO (Sc-16128-R), Anti-mouse β-actin (Sc-47778), and Anti-Mouse monoclonal Ubiquitin (K-48) (sc-271289) were purchased from Santa Cruz Biotechnology. BafA1 (CAS88899-55-2), Anti-Rabbit ATG5 (NB110-53818), and Anti-mouse SQSTM1/p62 (H00008878-M01) were purchased from Novus Biologicals. CQ (HR1258), MG132 (M8699), 5-nm Gold Goat anti-rabbit IgG (G7277), 10-nm Gold Goat anti-rabbit IgG (G7402), 10-nm Gold Goat anti-mouse IgG (G7777), 10-nm Gold Rabbit anti-goat IgG (G5402), and 5-nm Gold Goat anti-mouse IgG (G7527) were purchased from Sigma-Aldrich. NAC (S0077), Cell lysis buffer for Western and IP (P0013), and Enhanced BCA Protein Assay Kit (P0010) were purchased from Beyotime Biotechnology. Halt protease and phosphatase inhibitor cocktail (78440), FITC Chicken anti-goat IgG (A15964), FITC Donkey anti-mouse IgG (A15964), Alexa Fluor^®^ 488 Donkey anti-rabbit IgG (A15964, 21206), Anti-Mouse αM/CD11b (MA5-16604), and Anti-Mouse αM/CD11b (MA5-16528) were purchased from Thermo Scientific. HRP Goat anti-mouse IgG (SA00001-1), HRP Goat anti-rabbit IgG (SA00001-2), and HRP Rabbit anti-goat IgG (SA00001-4) were purchased from Proteintech.

### Preparation of the PA/BSA Complex Solution

Sodium palmitate (Sigma Aldrich, P9767) was dissolved in distilled water by heating at 70°C till completely dissolved. Simultaneously, 10% (wt/vol) FFA-free BSA solution was prepared at 55°C. The two solutions were mixed and coupled at 55°C for 10 min, made into 50 mM of PA/BSA complex stock solution.

### Autophagy Assays

#### Transmission Electron Microscopy

After being incubated for 2–4 h, neutrophils were pelleted and fixed with 4% glutaraldehyde in 0.1 M PBS overnight at 4°C. Subsequently, Postfixed in 1% osmium tetroxide was followed by dehydration with graded series of ethanol, infiltration, and embedding in SPI-PON 812 resin (SPI Supplies, West Chester, PA, USA). Ultrathin sections with a thickness of 65 nm were cut using a microtome Leica EM UC7 (Leica Microsystems Company, Wetzlar, Hessen, Germany) and poststained with 2% uranyl acetate for 10 min and 0.3% lead citrate for 10 min. The ultrathin sections were observed using a transmission electron microscope (H-7650, Kyoto, Japan).

#### LC3B Puncta Assays

Neutrophils (2×10^5^) were fixed on coverslips through cytospin. LC3B puncta assays were performed using an anti-LC3B antibody as described previously (22) and followed by a rapid nuclear dyeing with Hoechst 33258.

#### Evaluation of Autophagic Flux by Immunoblotting

During autophagy, more microtubule-associated light chain 3 (LC3)-I is esterified into LC3-II. In contrast, the accumulation of Sequestosome 1 (SQSTM1/p62) is degraded with autophagic substrates. Therefore, p62 negative indicates autophagy. The quantification of LC3B-II and p62 was used to evaluate the autophagic flux.

### Immunogold Electron Microscopy

Neutrophils were prepared for immunogold electron microscopy as previously described with minor modifications (23). Briefly, neutrophils were fixed in 4% paraformaldehyde and 0.5% glutaraldehyde at 4°C for 1.5 h, washed, scraped and pelleted, sectioned. The sections were soaked in pure water and then blocked in 3% skimmed milk in PBS for 30 min at room temperature. Subsequently, the sections were labeled with primary antibodies followed by secondary antibodies conjugated with protein A-gold. The sections were poststained with 2% uranyl acetate for 10 min before observation with a TEM (H-7650, Kyoto, Japan).

### Adhesion Assay

The 96-well plates were coated with Collagenase IV as substrates. Then, the cells were harvested after different administration (the *ex vivo* neutrophils were incubated with their own sera). Then, the neutrophils were labeled with Calcein AM (5 μM) for 30 min at 37°C. Subsequently, the labeled cells were washed, resuspended at 5.0 × 10^6^ cells/ml and then added to the 96-well plates. The plates were incubated at 37°C for 30 min. Nonadherent cells were removed with cold RPMI medium containing 1% FBS. The plate was scanned with a Tecan Infinite 200 PRO multifunctional microplate reader (TECAN, Männedorf, Switzerland), and the fluorescence of the adherent cells was measured by a Nikon fluorescence microscope (Nikon, Tokyo, Japan).

### Marker Assays

Granules can be distinguished on the basis of their morphological characteristics, such as size, shape, and electron density: AGs are large granules with high electron density; SGs are smaller than AGs and have lower electron densities; and GGs are the smallest granules in size and have the lowest electron densities. In addition, the three granule subunits are identifiable by their marker proteins: MPO is an AG marker, lactoferrin is an SG marker, and MMP-9 is a GG marker. These marker molecules were labeled using immunogold electron microscopy.

### Granule Quantification

The granules number were performed using the transmission electron microscopy. The sections were chosen randomly and then the granule number of each section was quantified based on their morphological characteristics, such as the size, shape, and electron density: AGs are the largest granules, with spherical and ellipsoid 2 kind of shape and high electron density; SGs are smaller than AGs, with a dumbbell shape and with lower electron density; GGs are the smallest granule in size, with a round shape and with the lowest electron density.

### Flow Cytometry

Surface analysis of CD11b and CD18 was performed using flow cytometry as previously described (24). Briefly, neutrophils were harvested, washed twice, and resuspended with ice-cold PBS containing 10% FBS and 1% sodium azide. Then, the neutrophils (2×10^5^) were incubated with primary antibody (1:100) at 4°C for 30 min. After being washed, the neutrophils were incubated with secondary antibody conjugated to FITC (1:200) at 4°C in the dark for 1 h. The mean fluorescence intensity of 10,000 neutrophils was assessed immediately using flow cytometry.

### Immunoprecipitation Assay

Neutrophils were harvested and incubated with cold 1×PBS containing 2 mM DFP on ice for 15 min. Cytosolic fractions of neutrophils were performed using a Cell Fractionation Kit (Danvers, MA, USA). Immunoprecipitation was performed from cytosolic fraction using a Pierce Crosslink Immunoprecipitation Kit (Thermo Scientific, MA, USA). One milligram of cytosolic fraction was precleared with 80 µl of the control agarose resin slurry for 1.5 h at 4°C. The primary antibodies were cross-linked to protein A/G plus agarose. The precleared lysate was added to the primary antibody-crosslinked resin in the column overnight at 4°C. The unbound proteins were washed away with IP lysis/wash buffer. Then, the immunoprecipitated proteins were eluted. The eluate concentrations were determined using the BCA Protein Assay Kit (Pierce, IL, USA). The protein complexes were analyzed by SDS-PAGE, and the gel was stained with Coomassie blue.

### Generation and Differentiation of the ATG5, p62, FGA, Hsc70, and TRIM21 Knockdown HL-60 Cell Lines

The lentiviral vectors for ATG5 knockdown (LV-GFP-shATG5), p62 knockdown (LV-GFP-shp62), and Hsc70 knockdown (LV-GFP-shHsc70) were purchased from GeneChem. The lentiviral vectors for FGA knockdown (LV-GFP-shFGA) and TRIM21 knockdown (LV-GFP-shTRIM21) were constructed by GeneChem. HL-60 cells were infected with lentiviral vectors at a MOI of 25 in the presence of 5 μg/ml polybrene. The HL-60 cells were differentiated to a neutrophil-like phenotype with a final concentration of 1.3% DMSO for 6 days.

### Shotgun Analysis

Endogenous CD11b and CD18 were immunoprecipitated using a Pierce Crosslink Immunoprecipitation (IP) Kit (Thermo Scientific, 26147). Approximately 30 μg of IP complexes of CD11b and CD18 was performed by Shotgun Analysis as previously described. 30 MS/MS spectra were searched using MASCOT engine (version 2.2, Matrix Science) embedded into Proteome Discoverer 1.3 against the Uniprot Human database (88,611 sequences, downloaded on December 28, 2015). For protein identification, the following parameters were selected: Peptide mass tolerance: 20 ppm, MS/MS tolerance: 6 ppm, Enzyme: Trypsin, Max Missed Cleavages: 2, Fixed modifications: Carbamidomethyl (C), Dynamical modifications: Oxidation (M) and GlyGly (K), peptides FDR ≤ 0.01, protein FDR ≤ 0.01, and Filter by score ≥ 20.

### Protein Oxidation Detection

Protein oxidation was performed using the OxyBlot protein oxidation detection kit (Millipore, S7150) according to the manufacturer’s instructions (25). Briefly, the IP complexes of CD11b and CD18 were denatured by adding 12% SDS, derivatized by adding 1 × DNPH solution, incubated at room temperature for 15 min, and neutralized with neutralization solution and 2-mercaptoethanol at a final concentration of 0.74 M. The levels of DNP were detected using immunoblotting.

### Statistical Analysis

Statistical analysis was carried out using PRISM 6 (GraphPad). The unpaired *t*-test (when comparing two groups), one-way ANOVA test (when comparing with a single group), and two-way ANOVA (when comparing multiple factors between two groups) were used in this study as indicated in the figure legends. Individual *p*-values are indicated in the figures, with no data points excluded from statistical analysis.

## Results

### Fatty Liver Dairy Cow Neutrophils Have Enhanced Autophagy and Vacuolation Coinciding With Adhesion Deficiency

**Figure 1:**
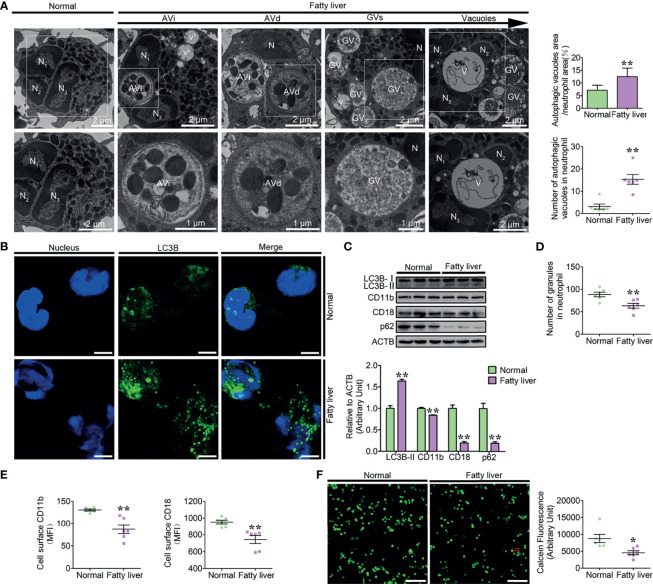
Increased autophagy, vacuolation, and adhesion deficiency existed in fatty liver dairy cow neutrophils. **(A)** Representative transmission electron micrographs of normal and fatty liver neutrophils. White arrows indicate autophagic vacuoles (AVi, AVd, GVs, and vacuoles). N (N1, N2, and N3), nucleus. Scale bars as indicated. The area ratio of autophagic vacuoles to neutrophils and the number of autophagic vacuoles in neutrophils were determined (*n* = 6). Data represent the mean ± s.e.m. (**p < 0.01 *versus* the control group; Significance calculated using *t*-test). **(B)** Autophagy levels were evaluated using confocal microscopy in neutrophils of normal and fatty liver cows. Neutrophils were stained with anti-LC3B antibody and nuclear DNA was stained with Hoechst 33258. Scale bar, 5 μm. **(C)** Immunoblot for LC3B, CD11b, CD18, and p62 in normal and fatty liver neutrophils. ACTB was used as a loading control (*n* = 3). Data represent the mean ± s.e.m. (**p < 0.01 *versus* the control group; significance calculated using two-way ANOVA). **(D)** The number of granules in neutrophils (*n* = 6) of normal and fatty liver cows. Data represent the mean ± s.e.m. (**p < 0.01 *versus* the control group; significance calculated using *t*-test). **(E)** Surface expression of CD11b and CD18 on normal and fatty liver cow neutrophils (*n* = 6). Surface expression of CD11b and CD18 was assessed by flow cytometry analysis (*n* = 6). MFI, mean fluorescence intensity. Data represent the mean ± s.e.m. (*p < 0.05 and **p < 0.01 *versus* the control group; significance calculated using *t*-test). **(F)** Representative fluorescence micrograph images (left) and fluorescence microplate analysis (right) of normal and fatty liver cow neutrophils (*n* = 6). Scale bar, 400 μm. Data represent the mean ± s.e.m. (*p < 0.05 *versus* the control group; significance calculated using *t*-test).

Due to the dysregulation of the breakdown and synthesis metabolism of fat in the liver of fatty liver dairy cows, excessive free fatty acids that cannot be completely metabolized are released into the blood, resulting in an abnormally high concentration of PA in the blood. Fatty liver dairy cow neutrophils are exposed to the high blood PA, which may strongly induce neutrophil autophagy, further triggering the degradation of the damaged granules, thus causing neutrophil vacuolation. Moreover, these intracellular granules are distributed with many αMβ2 integrins, which are implicated in the regulation of neutrophil firm adhesion *via* the mechanism of granule mobilization (26). So, we hypothesized that high blood PA should induce autophagy-dependent granule degradation, leading to neutrophil vacuolation and adhesion deficiency in dairy cows with fatty liver. Therefore, normal (*n* = 12) and fatty liver dairy cows (*n* = 12) were selected to explore the autophagic flux and the levels of vacuolation and adhesion in neutrophils of these cows. Fatty liver dairy cows were diagnosed by both serologic examination and hepatic H&E staining (**Supplementary Figure S1**), and the biochemical parameters and clinical characteristics of normal and fatty liver cows are listed in (**Table 1**). Our findings showed that enhanced autophagy, increased vacuolation, and adhesion deficiency existed in neutrophils of dairy cows with fatty liver. To facilitate the understanding of the role of autophagy in neutrophil vacuolation, four consecutive stages of autophagic vacuoles in fatty liver neutrophils were defined based on the degree of degradation of intracellular granules: early autophagic vacuoles (AVi), degradative autophagic vacuoles (AVd), glycogen vacuoles (GVs), and vacuoles (27–30). The four stages of autophagic vacuoles were observed in fatty liver dairy cow neutrophils, and the number of autophagic vacuoles, the ratio of the neutrophil vacuolation area (**Figure 1A**), the accumulation of LC3B puncta (**Figure 1B**), and the lipidation levels of LC3B were markedly increased and the accumulation of p62 was markedly decreased (**Figure 1C**) in fatty liver dairy cow neutrophils. These results indicate that autophagy activity is upregulated in fatty liver dairy cow neutrophils. At the AVi and AVd stages, numerous granules were engulfed by autophagic vacuoles and were further degraded into vacuoles (**Figure 1A**). Expectedly, the number of granules in fatty liver dairy cow neutrophils was significantly reduced (**Figure 1D**). These intracellular granules of neutrophils contain many CD11b and CD18, which could be integrated into plasma membrane *via* the degranulation and play an important role in neutrophil adhesion (1, 31). So, the total protein levels and surface expression of the CD11b and CD18 were performed. As expected, the total protein and surface levels of CD11b and CD18 were significantly decreased in fatty liver dairy cow neutrophils (**Figures 1C, E** and **Supplementary Figure S2**). These results implied that the two adhesion-associated molecules had been degraded together with granules. CD11b and CD18 have the highest amount of β2 integrins, which are required for neutrophil firm adhesion. Therefore, the adhesion assay was employed to assess neutrophil adhesion. Results showed that fatty liver neutrophil adhesion was markedly decreased (**Figure 1F**), suggesting that neutrophils from dairy cows with fatty liver had increased autophagy, vacuolation, and adhesion deficiency.

### PA-Triggered Autophagy-Dependent Degradation of the Granules and Vacuolation in Dairy Cow Neutrophils

**Figure 2:**
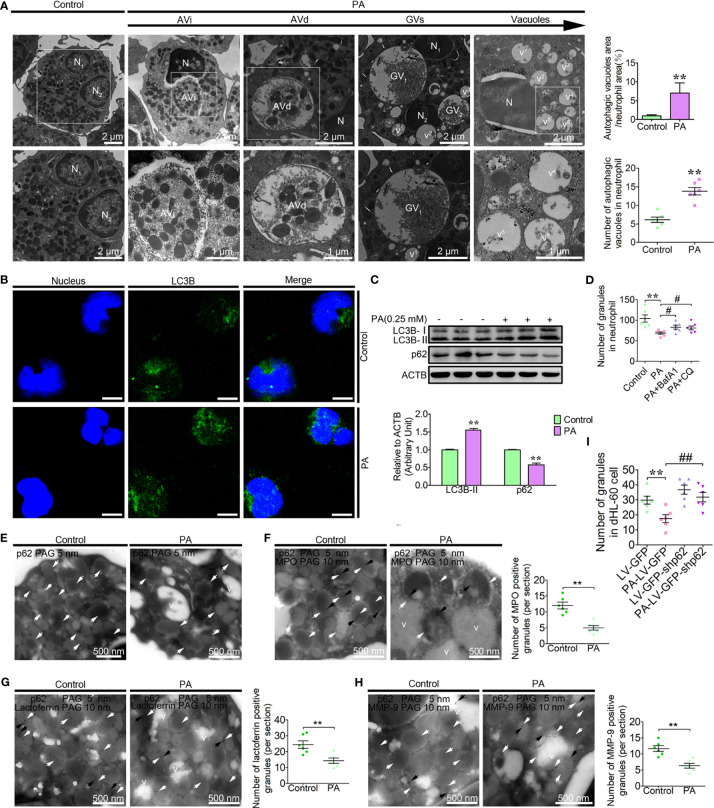
PA triggers autophagy-dependent degradation of the granules and vacuolation in dairy cow neutrophils. **(A)** Representative transmission electron micrographs of control and PA (0.25 mM)-treated neutrophils (left). White arrows indicate autophagic vacuoles (AVi, AVd, glycogen vacuoles, and vacuoles). N (N1, N2, N3…), nucleus. Scale bars as indicated. The area ratio of autophagic vacuoles to neutrophils and the number of autophagic vacuoles in neutrophils were determined (right, *n* = 6). Data represent the mean ± s.e.m. (**p < 0.01 *versus* the control group; significance calculated using *t*-test). **(B)** Autophagy levels were evaluated using confocal microscopy in control and PA-treated neutrophils. Neutrophils were stained with anti-LC3B antibody and nuclear DNA was stained with Hoechst 33258. Scale bar, 5 μm. **(C)** Immunoblot for LC3B and p62 in control and PA-treated bovine neutrophils. ACTB was used as a loading control (*n* = 3). Data represent the mean ± s.e.m. (**p < 0.01 *versus* the control group; significance calculated using two-way ANOVA). **(D)** The number of granules in PA-treated neutrophils (autophagy pathway was blocked using BafA1, CQ or not, *n* = 6). Data represent the mean ± s.e.m. (**p < 0.01 *versus* the control group, # < 0.05 and *versus* the PA-treated group; Significance calculated using one-way ANOVA). **(E)** Immunogold electron micrograph showing the localization of p62 in control and PA-treated bovine neutrophils. **(F)** P62-mediated PA-triggered autophagy-dependent degradation of AGs, SGs, and GGs in bovine neutrophils. Partial view of immunogold electron micrograph showing the colocalization of p62 with the AG marker MPO **(F)**, SG marker lactoferrin **(G)**, and GG marker MMP-9 **(H)** in control and PA-treated BPMNs. White arrows (5-nm gold grains) indicate p62. Black arrows (10-nm gold grains) indicate MPO, lactoferrin, and MMP-9. v (v1, v2, v3…), vacuoles. **(I)** The number of granules in control and PA-treated dHL-60 cells (infected with LV-GFP-shp62) was determined (*n* = 6). Data represent the mean ± s.e.m. (**p < 0.01 *versus* the control group, ^##^ *p* < 0.01 and *versus* the PA-treated group; significance calculated using one-way ANOVA).

Based on the clinical parameters and *ex vivo* results of the high blood NEFAs dairy cow, we found that the increased autophagy and vacuolation of neutrophils correlated with high blood PA (≥0.12 mM) levels. The increased vacuolation of fatty liver dairy cow might be associated with autophagic granule degradation. To investigate the role of PA in the autophagy-dependent vacuolation of neutrophils in dairy cows with fatty liver, neutrophils were treated with the simulated pathological concentration PA (0.25 mM). Consistent with the *ex vivo* findings, the four stages of autophagic vacuoles were observed in PA-treated neutrophils (**Figure 2A**). The number of autophagic vacuoles and the ratio of autophagic vacuole area to neutrophil area were significantly increased in PA-treated neutrophils compared to control neutrophils (**Figure 2A**). Furthermore, the accumulation of the LC3B puncta and the lipidation levels of LC3B were significantly increased in PA-treated neutrophils, while p62 was significantly decreased (**Figures 2B, C**). The accumulation of granules significantly decreased in PA-treated dairy cow, but when autophagy was blocked by bafilomycin A (BafA1) and hydroxychloroquine sulfate (CQ), the degradation of granules induced by PA was significantly lower (**Figure 2D**). Neutrophils were also stimulated with fMLP to further induce degranulation upon PA treatment. No difference was observed in granule numbers in PA-treated neutrophils when the degranulation was further induced (**Supplementary Figure S3**). These results suggest that PA-induced autophagy plays a dominant role in neutrophil granule homeostasis. These results show that PA strongly enhances neutrophil autophagy and autophagy-dependent granule degradation, leading to increased dairy cow neutrophil vacuolation.

p62, as one of the most important autophagic receptors, targets damaged mitochondria to lysosomes for degradation (32). To investigate whether p62-mediated the degradation of these granules through autophagy, PA-treated neutrophils were labeled with p62 and observed by electron microscopy. The p62 grains were mainly located on the granules in the control neutrophils (**Figure 2E**). However, the p62 grains were predominantly on the AVi and AVd but rarely on the vacuoles in PA-treated neutrophils (**Figure 2E**). These results suggest that p62 on the vacuoles might be degraded by autophagy. As mentioned, neutrophils are equipped with three granule subpopulations, including AGs, SGs, and GGs, which can be roughly distinguished according to their size, electron density, and marker proteins. To further investigate whether p62 targets the three granule subunits for degradation. The colocalization of p62 with AGs marker protein, MPO, SGs marker protein, lactoferrin and GGs marker protein, MMP-9 were performed as *Marker Assays*. Data showed that p62 colocalized with MPO, lactoferrin, and MMP-9, which are the representative markers for AGs, SGs, and GGs, respectively (**Figures 2F–H**). These results show that p62 might mediate the degradation of the AGs, SGs, and GGs triggered by PA. To further confirm that p62 indeed mediates the degradation of the granules, neutrophil-like differentiated HL-60 cells (dHL-60) were infected with lentivirus to knock down p62. p62 knockdown attenuated the decrease of granules induced by PA in dHL-60 (**Figure 2I**). These results indicate that p62 mediates autophagy-induced degradation of the intracellular AGs, SGs, and GGs, thereby causing the decrease of granule levels and vacuolation in PA-treated dairy cow neutrophils.

### PA-Induced Autophagy Reduced Neutrophil Adhesion

**Figure 3:**
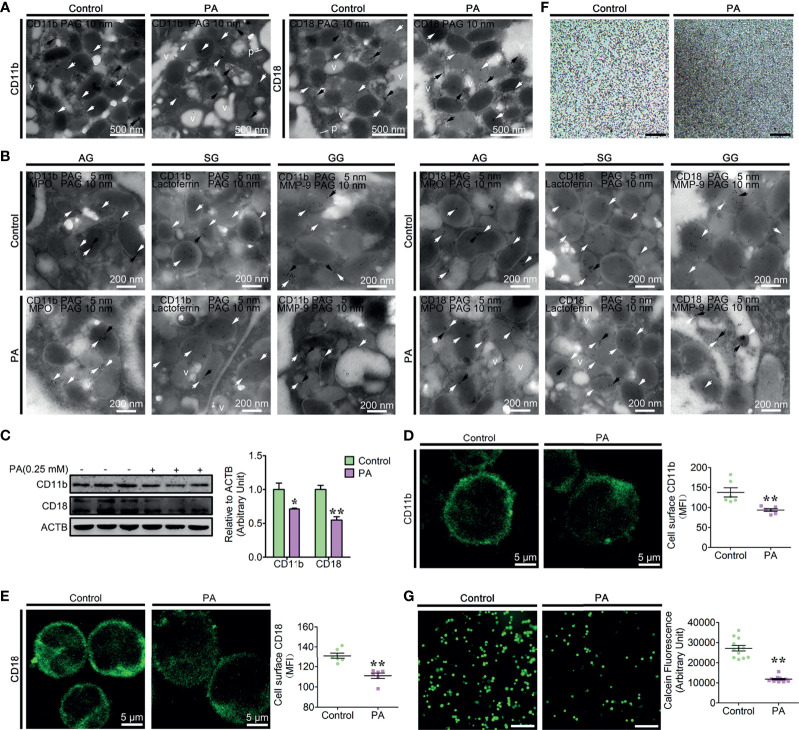
Autophagy-mediated PA triggered the decrease of dairy cow neutrophil adhesion **(A)** Immunogold electron micrograph showing the localization of CD11b in control and PA-treated bovine neutrophils. White arrows show gold grains on the granules, black arrows show gold grains on AVi and AVd, and v shows gold grains on vacuoles. N, nucleus. p, plasma membrane. Scale bars as indicated. **(B)** Portion of immunogold electron micrographs of control and PA-treated bovine neutrophils labeled for MPO, lactoferrin, or MMP-9 (10-nm gold grains, black arrows) and then labeled for CD11b and CD18 (5-nm gold grains, white arrows). Scale bars as indicated. **(C)** Immunoblot for the total protein expression of CD11b and CD18 in control and PA-treated bovine neutrophils (*n* = 3). Data represent the mean ± s.e.m. (*p < 0.05 and **p < 0.01 *versus* the control group; significance calculated using two-way ANOVA). **(D)** Representative immunofluorescence images and flow cytometry analysis of the surface expression of CD11b on control and PA-treated bovine neutrophils (*n* = 6). Data represent the mean ± s.e.m. (**p < 0.01 *versus* the control group; significance calculated using *t*-test). **(E)** Representative immunofluorescence images and flow cytometry analysis of the surface expression of CD18 on control and PA-treated bovine neutrophils (*n* = 6). Data represent the mean ± s.e.m. (**p < 0.01 *versus* the control group; significance calculated using *t*-test). **(F)** Representative light microscopy images of control and PA-treated bovine neutrophils. Scale bar, 2 mm. **(G)** Representative fluorescence micrograph images (left) and fluorescence microplate analysis (right) of normal and fatty liver bovine neutrophils (*n* = 6). Scale bar, 400 μm. Data represent the mean ± s.e.m. (**p < 0.01 *versus* the control group; significance calculated using *t*-test).

β2 integrins are essential for the neutrophil firm adhesion, trans-endothelial migration, and the recruitment of neutrophils to the sites of inflammation (9, 10). Based on the *ex vivo* results, high blood PA and neutrophil adhesion deficiency coexist in the fatty liver dairy cows, which indicates that PA is associated with the decrease of neutrophil adhesion. To confirm this hypothesis, we first investigated the role of PA in the dynamic distribution of CD11b and CD18; control and PA-treated neutrophils were labeled with CD11b and CD18. In control neutrophils, CD11b and CD18 grains were observed on the granules with different size and electron density, cytoplasmic matrix and plasma membrane (**Figure 3A**), whereas in PA-treated neutrophils, the CD11b and CD18 grains decreased and primarily located on the AVi and AVd, but sparsely distributed on vacuoles (**Figure 3A**). These results suggested that the expression of the CD11b and CD18 decreased and CD11b and CD18 on the granules might be degraded together with granules by autophagy induced by PA. We next checked whether CD11b and CD18 were also distributed on AGs, SGs, and GGs using IEM double labeling (CD11b and CD18 colocalized with MPO, lactoferrin and MMP-9, respectively) and the morphological analysis of the three granule subunits. The IEM double-labeling results showed that CD11b and CD18 grains were colocalized with MPO on the large and highly electron-dense AGs, were colocalized with lactoferrin on the smaller and less electron-dense SGs, and were colocalized with MMP-9 on the smallest and least electron-dense GGs, but fewer or none were located on vacuoles (**Figure 3B**). These results indicate that AGs, SGs, and GGs were distributed with CD11b and CD18 and PA triggers CD11b and CD18 on the three granule subunits autophagy-dependent degradation. To further confirm that PA triggered the degradation of CD11b and CD18, the accumulation of the two proteins in total proteins was performed by WB and the surface expression of CD11b and CD18 using flow cytometry and immunofluorescence. The results indicated that PA significantly triggered the decrease of the total protein levels of CD11b and CD18 and the surface expression of CD11b and CD18 compared to control neutrophils (**Figures 3C–E** and **Supplementary Figure S4**). Based on transmission light microscopy, the control neutrophils adhered to the plates evenly and tightly (**Figure 3F**). On the contrary, PA-treated neutrophils adhered to the plates loosely, and several cell lumps were observed floating in the medium (**Figure 3F**). The above results motivated us to speculate that PA might trigger the decrease of the dairy cow neutrophil adhesion. Therefore, we further determined the adhesion of PA-treated neutrophils as described in adhesion array. Expectantly, the adhesion array results showed that the adhesion of PA-treated neutrophils decreased sharply compared to that of control neutrophils (**Figure 3G**). This was not due to a cytotoxic effect of PA on neutrophils at the concentration used in our experiments (0.25 mM) (**Supplementary Figure S5**). Taken together, these results demonstrate that the AG, SG, and GGs are distributed with CD11b and CD18 and PA triggers CD11b and CD18 granule-dependent degradation *via* autophagy, thereby further significantly decreasing neutrophil adhesion in high blood PA dairy cow.

### PA Triggered the Decrease of the CD11b and CD18 Through the Autophagy-Dependent Degradation Pathway

**Figure 4:**
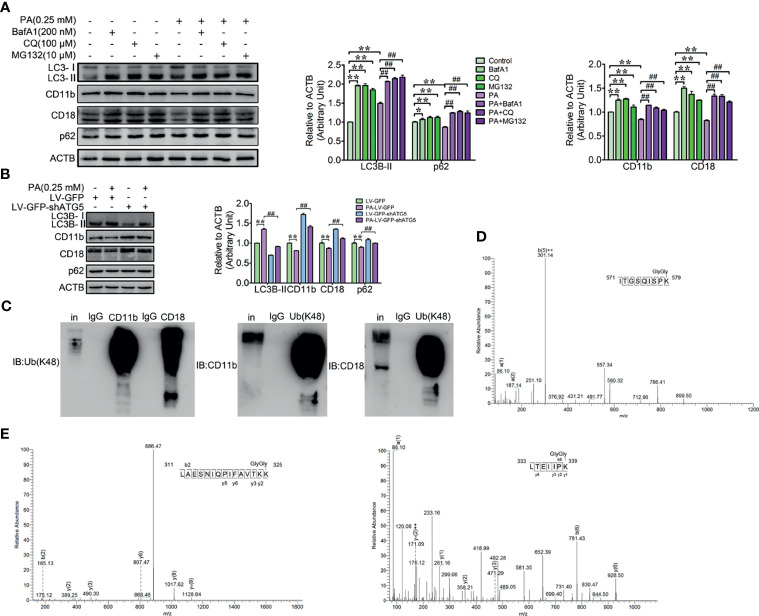
Autophagy-mediated PA triggered the degradation of ubiquitinated CD11b and CD18 in dairy cow neutrophils. **(A)** Immunoblot and quantitative analysis for LC3B, p62, CD11b, and CD18 in control and PA-treated bovine neutrophils (treated or not treated with BafA1, CQ or MG132). The protein degradation was blocked by BafA1, CQ, or MG132 in control and PA-treated bovine neutrophils. Quantitative analysis of LC3B, p62, CD11b, and CD18 (*n* = 3) was performed. Data represent the mean ± s.e.m. (*p < 0.05 and **p < 0.01 *versus* the control group, ^##^ *p* < 0.01 *versus* the PA-treated group; significance calculated using two-way ANOVA). **(B)** Immunoblot and quantitative analysis for LC3B, p62, CD11b, and CD18 in control and PA-treated dHL-60 cells (infected or not infected with LV-GFP-shATG5). Data represent mean ± s.e.m. Note: (**p < 0.01 *versus* the control group, ^##^ *p* < 0.01*versus* the PA-treated group). Data represent the mean ± s.e.m. (**p < 0.01 *versus* the control group, ^##^ *p* < 0.01 *versus* the PA-treated group; significance calculated using two-way ANOVA). **(C)** Reciprocal co-IP of CD11b and CD18 with polyubiquitin chains (Lys48). **(D)** Determination of ubiquitination sites in the CD11b fragment (571-ITGSQISPK (GlyGly)-579) by MS/MS spectra. **(E)** Determination of ubiquitination sites in the CD18 fragments (311-LAESNIQPIFAVTKK(GlyGly)-325, 333-LTEIIPK (GlyGly)-339) by MS/MS spectra.

Both the *ex vivo* and *in vitro* results have demonstrated that PA strongly triggers autophagy and the decrease of CD11b and CD18 of dairy cow neutrophils. In order to investigate whether autophagy directly contributes to the decrease of the CD11b and CD18, autophagy blocker BafA1, CQ, and ubiquitination pathway inhibitor MG132 were employed to block autophagy pathway. We found that the decrease of the CD11b and CD18 triggered by PA was significantly attenuated after the autophagy was blocked by BafA1, CQ, and MG132 (**Figure 4A**). To further confirm that the decrease of the CD11b and CD18 is indeed mediated by autophagy, ATG5 knockdown lentivirus was used to infect the dHL-60, and the accumulation of CD11b and CD18 was confirmed by immunoblotting. The results again indicated that ATG5 knockdown rescued the decrease of the CD11b and CD18 induced by PA (**Figure 4B**). From these results, we reasoned that the degradation of the CD11b and CD18 indeed *via* autophagy at least partly. Ubiquitination is a prerequisite for protein degradation by autophagy (33). So, the ubiquitination of the CD11b or CD18 was detected by immunoblotting and mass spectrometry assay. The results showed that CD11b and CD18 could be modified by polyubiquitination (**Figure 4C**); 579 K of CD11b, and 325 K and 339 K of CD18 were ubiquitinated directly (**Figures 4D, E**). In summary, 579 K of CD11b, and 325 K and 339 K of CD18 were direct ubiquitinated. PA triggers the degradation of CD11b and CD18 *via* the autophagy pathway and then decreases the accumulation of the CD11b and CD18.

### FGA, Hsc70, and TRIM21-Mediated PA Induced the Cytosolic Oxidized-Ubiquitinated CD11b and CD18 Degradation

**Figure 5:**
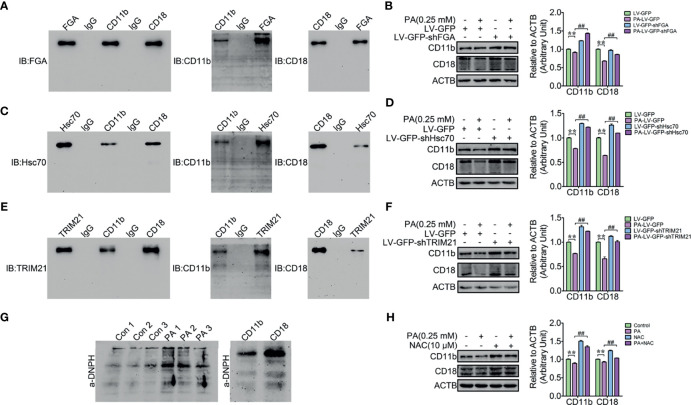
FGA, Hsc70, and TRIM21-mediated PA induced the cytosolic oxidized CD11b and CD18 degradation. **(A)** Reciprocal co-IP of CD11b and CD18 with FGA in dHL-60 cells. **(B)** Knockdown of FGA blocked the degradation of CD11b and CD18 in dHL-60 cells (*n* = 3). Data represent the mean ± s.e.m. (**p < 0.01 *versus* the control group, ^##^ *p* < 0.01 and *versus* the PA-treated group; significance calculated using one-way ANOVA). **(C)** Reciprocal co-IP of CD11b and CD18 with Hsc70 in dHL-60 cells. **(D)** Knockdown of Hsc70 blocked the degradation of CD11b and CD18 in dHL-60 cells (*n* = 3). Data represent the mean ± s.e.m. (**p < 0.01 *versus* the control group, ^##^ *p* < 0.01 and *versus* the PA-treated group; significance calculated using one-way ANOVA). **(E)** Reciprocal co-IP of CD11b and CD18 with TRIM21 in dHL-60 cells. **(F)** Knockdown of TRIM21 blocked the degradation of CD11b and CD18 in dHL-60 cells (*n* = 3). Data represent the mean ± s.e.m. (**p < 0.01 *versus* the control group, ^##^ *p* < 0.01 and *versus* the PA-treated group; significance calculated using one-way ANOVA). **(G)** Immunoblot for the analysis of CD11b and CD18 oxidation. **(H)** NAC relieved PA-triggered by the degradation of CD11b and CD18. Data represent the mean ± s.e.m. (**p < 0.01 *versus* the control group, ^##^ *p* < 0.01 and *versus* the PA-treated group; significance calculated using one-way ANOVA).

A fraction of cytoplasmic ubiquitinated α5β1 integrins could be degraded *via* the microautophagy in a fibronectin-dependent manner (34). However, the underlying degradation mechanism of the cytoplasmic β2 integrin is unknown. Therefore, the IP complexes of CD11b and CD18 were identified by a shotgun analysis. A total of 210 proteins were identified as interacting with CD11b (**Supplementary Table S1**), and 260 proteins were identified as interacting with CD18 (**Supplementary Table S2**). Importantly, a lot of the ubiquitinated peptides of fibrinogen alpha chain (FGA) (Accession: P02672), Heat shock cognate 71 kDa protein (Hsc70) (P19120), and tripartite motif-containing protein 21 (TRIM21) (Q7YRV4) commonly interacted with both CD11b and CD18 (**Figures 5A, C, E**). FGA is a β2 integrin ligand protein, Hsc70 is a molecular chaperone protein, and TRIM21 is a E3 ubiquitin ligase. These results motivated us to investigate whether the cytoplasmic ubiquitinated CD11b and CD18 could be ubiquitinated by TRIM21 and then degraded by microautophagy in an FGA-dependent manner or by Hsc70-mediated chaperone-mediated autophagy (CMA). Therefore, FGA knockdown lentivirus, Hsc70 knockdown lentivirus, and TRIM21 knockdown lentivirus infected dHL-60 cells to check whether FGA, Hsc70, and TRIM21 mediated the degradation of CD11b and CD18, and PA was used to induce the degradation of CD11b and CD18. Immunoblotting results showed that FGA knockdown (**Figure 5B**), Hsc70 knockdown (**Figure 5D**), and TRIM21 knockdown (**Figure 5F**) all attenuated the degradation of CD11b and CD18 triggered by PA. The cytoplasmic light oxidized–ubiquitinated protein aggregates and severe oxidized–ubiquitinated aggregates were mainly degraded *via* the ubiquitin–proteasome pathway and the CMA pathway, respectively (35, 36). So, the oxidative modification of CD11b and CD18 was detected by the Protein Oxidation Detection Kit. Findings showed that both CD11b and CD18 could be oxidized (**Figure 5G**) and ROS scavenger NAC relieved the degradation of CD11b and CD18 triggered by PA (**Figure 5H**). Taken together, FGA, Hsc70, and TRIM21 mediate the degradation of the cytoplasmic oxidized–ubiquitinated CD11b and CD18 *via* autophagy.

## Discussion

Granules are essential for neutrophils to fulfill their functional innate immunity roles, including early activation, migration to injured tissues, and the destruction of the intruding microorganisms (37). Autophagy, a lysosome-dependent degradation mechanism, plays a pivotal role in maintaining cellular homeostasis by engulfing unnecessary cytosolic cargoes or damaged organelles for degradation. Autophagy can eliminate damaged mitochondria, which has been well documented in energy cells (14). Neutrophils are densely filled with the three granule subunits, namely, AGs, SGs, and GGs. Here, we found that high blood PA triggered autophagy-dependent degradation of the three neutrophil granule subunits, leading to an increased vacuolation of neutrophils in dairy cows with fatty liver. Additionally, β2 integrins are essential for the firm adhesion and transepithelium migration of neutrophils to the inflamed sites. Importantly, two β2 integral cell-surface proteins CD11b and CD18 on the three granule subunits were degraded together with granules by autophagy, thereby causing the adhesion deficiency of neutrophil in dairy cows with fatty liver. Moreover, a fraction of cytoplasmic oxidized–ubiquitinated CD11b and CD18 could also be mediated by FGA, Hsc70, and TRIM21 for degradation. Knockdown of ATG5 restored neutrophil vacuolation levels, increasing the accumulation of intracellular granules as well as CD11b and CD18 levels in dHL-60 cells. These observations uncover an unreported role of autophagy in maintaining the neutrophil granule homeostasis and regulating the migration of neutrophil and provide a new perspective to explain the reason of the innate immunosuppression of the dairy cows with fatty liver.

Increased vacuolation in neutrophils is viewed as an indicator of the immune activation (38). Vacuolated neutrophils are observed in ethanol toxified and septicemia patients due to the changes in their blood constituents. Fatty liver is a highly prevalent lipid metabolism disorder of lactating dairy cows with decreased reproductive performance and immune status. The feed intake of dairy cows with fatty liver is decreased, while the energy expenditure of lactating increased; fat mobilization is activated to meet this process, and a lot of NEFAs were produced in the liver. Fatty live dairy cows suffer from liver injury and lipid metabolism disorder; the NEFAs that cannot be completely metabolized will be released into the blood. Neutrophils are explored to the high blood NEFAs, which might induce autophagy-dependent granule degradation and vacuolation. In addition, the granular proteins were totally exhausted, suggesting increased vacuolization of neutrophils and dysfunction in dairy cows with fatty liver.

Autophagy receptor p62 mediates the degradation of the damaged mitochondria in energy cells (14). Neutrophils are densely equipped with AGs, SGs, and GGs. In this study, most p62 colocalized with MPO, lactoferrin, and MMP-9 on AGs, SGs, and GGs, respectively, or located on the AVi and AVd of neutrophil autophagic vacuoles. Little to no signal was observed on the glycogen vacuole and vacuole stages, which indicated that p62 might deliver the damaged AGs, SGs, and GGs to lysosomes for degradation *via* autophagy. Interestingly, neutrophil granules are analogous to classic lysosomes (1, 39). Due to the metabolism disorder of the liver NEFAs in dairy cows with fatty liver, excessive NEFAs are released into the blood. Neutrophil exposed to the high blood NEFAs might trigger the membrane permeabilization of the granules (**Supplementary Figure S6**). The lysosomal membrane permeabilization (LMP) of the neutrophils will change and the integrity of lysosomes is lost (40). The damaged lysosomes can be eliminated by lysophagy (41). Moreover, lysosomes can fusion with autophagosomes, which process further damaged lysosomes, and lysosomal proteins are released into the cytoplasm. Degranulation is the process of regulated exocytosis of these lysosome-like granules. Autophagy abrogation inhibits degranulation (16). Whether the fusion of the granules with autophagosomes plays a role in the regulation of degranulation warrants further investigation.

The orchestrated degradation of β1 integrins is required for proper cancer cell migration and has been well documented (34). Autophagy decreases the matrix adhesion to facilitate tumor metastasis by degrading β1 integrins (34). By inhibition of autophagy, cancer cells will firmly adhere *in situ* (42). Consistently, autophagy blocker BafA1, CQ, the ubiquitination pathway inhibitor MG132, and ATG5 knockdown increased the accumulation of the CD11b and CD18 in dairy cow neutrophils or dHL-60 cells. These results confirm that autophagy not only controls tumor metastasis but also controls the immune cell migration through promoting the turnover of β2 integrins. Moreover, autophagy blocking, p62 knockdown (**Figure 1** and **Supplementary Figure S7**), or ATG5 knockdown relieved the degradation of the neutrophil granules and vacuolation, as well as the granule-dependent degradation of CD11b and CD18 in dairy cow neutrophils. These results suggest that autophagy might mediate neutrophil vacuolation and adhesion deficiency in a granule-dependent manner. The β2 integrin heterodimer has three conformations: bent, extend, and open states. The bent form cannot bind to ligands, whereas the extend form possesses intermediate ligand binding capacity and the open form has full avidity for ligand binding (43). The small GTPase Rap1, a critical β2 integrin regulator (44), is also present on three granule subunits and they might be degraded together with the granules (1). These findings suggest that autophagy might control neutrophil adhesion by both degrading β2 integrins directly and reducing the activity of β2 integrins by facilitating Rap1 degradation.

Ubiquitination is a prerequisite for protein degradation (33). Many ubiquitination forms involve the degradation of membrane proteins, including direct ubiquitination (direct ubiquitination of lysine residues) and indirect ubiquitination (ligand-dependent, dimer-dependent, high-affinity binding protein-dependent, chaperone molecular-dependent, and organelle-dependent ubiquitination). Even though CD11b and CD18 can be modified by polyubiquitination and 579 K of CD11b, and 325 K and 339 K of CD18 are ubiquitinated directly, the underlying degradation mechanism of CD11b and CD18 is still unclear. Therefore, the interacting proteins of CD11b and CD18 were identified *via* Shotgun. A total of 210 proteins can interact with CD11b and 260 proteins can interact with CD18. p62 can target the polyubiquitinated aggregates and deliver them to lysosomes for degradation (45). However, no peptides of p62 are identified, indicating that the cytoplasmic CD11b and CD18 cannot form polyubiquitinated aggregates and are targeted by p62 for degradation or the abundance of p62 is too low to be identified. Importantly, many ubiquitination peptides of β2 integrin ligand protein, FGA (P02672), molecular chaperone protein, Hsc70 (P19120) and E3 ubiquitinated ligase, TRIM21 (Q7YRV4) were identified to interact with CD11b and CD18 simultaneously. We utilized dHL-60 cells that were infected with FGA, Hsc70, and TRIM21 knockdown lentiviruses. FGA, Hsc70, and TRIM21 knockdown could alleviate PA-triggered degradation of CD11b and CD18 in dHL-60 cells. The cytoplasmic ubiquitinated β1 integrins can be degraded by the endosomal microautophagy in a ligand (fibronectin)-dependent manner (34). The ubiquitin–proteasome pathway mediated the degradation of light oxidized–ubiquitinated protein aggregates and the CMA pathway mediated the degradation of severe oxidized–ubiquitinated aggregates (35, 36). These results suggested that a fraction of cytoplasmic oxidized–ubiquitinated CD11b and CD18 severe aggregates is degraded by chaperone Hsc70-mediated molecular chaperone autophagy, and a fraction of cytoplasmic oxidized–ubiquitinated CD11b and CD18 light aggregates are modified by TRIM21 and then degraded by the ubiquitinated proteasome pathway.

In summary, this study reveals a role for autophagy in regulating the three granule subtypes, which are encapsulated by autophagosomes and then undergo the AVi, AVd, GVs, and vacuole stages, eventually leading to increased neutrophil vacuolation in dairy cow with high blood PA. Importantly, we also uncovered the role of extensive autophagy in the regulation of neutrophil migration by the orchestrated degradation of β2 integrins. Moreover, proteins related to phagocytosis, chemotaxis, cytoskeletal remodeling, and apoptosis are also found on granules (1), suggesting that autophagy may be involved in these biological functions. Since, an increased neutrophil vacuolation exists in ethanol toxicity, septicemia patients and high blood PA dairy cow, so further research is needed to investigate in the bacteremia, toxemia, diabetes, diabetic ketosis, hyperketonemia and hyperlipidemia patients. Understanding the mechanism and physiological meaning of neutrophil autophagy is fundamental to the development of novel therapeutic strategies against the above disease-induced innate immune deficiency.

## Data Availability Statement

The datasets presented in this study can be found in online repositories. The names of the repository/repositories and accession number(s) can be found in the article/**Supplementary Material**.

## Ethics Statement

The animal study was reviewed and approved by the Ethics Committee on the Use and Care of Animals at Jilin University.

## Author Contributions

ZP: conceptualization, methodology, investigation, and writing—original draft. CZ, and XD: formal analysis, writing—review and editing, and resources. YY, YL, YS, and BF: formal analysis, writing—review and editing. YMZ: formal analysis and data curation. XQ, YYZ, XbL, ZW, and XwL: project administration, writing—review and editing, and supervision. GL: conceptualization, methodology, writing—review and editing, and supervision. All authors contributed to the article and approved the submitted version.

## Conflict of Interest

The authors declare that the research was conducted in the absence of any commercial or financial relationships that could be construed as a potential conflict of interest.

## Publisher’s Note

All claims expressed in this article are solely those of the authors and do not necessarily represent those of their affiliated organizations, or those of the publisher, the editors and the reviewers. Any product that may be evaluated in this article, or claim that may be made by its manufacturer, is not guaranteed or endorsed by the publisher.

## Supplementary Material

Supplementary Figure 1 Representative hematoxylin and eosin (H&E)-stained images of liver tissues in normal and fatty liver cows.

Supplementary Figure 2 Flow cytometry images of the surface expression of CD11b and CD18 on Normal and Fatty liver dairy cow neutrophils.

Supplementary Figure 3 The number of granules in normal and PA-treated neutrophils with or without using fMLP to induce degranulation. Control and PA-treated neutrophils were cultured for 6h and then stimulated with 1 μM fMLP for 30 min to induce degranulation and then were collected to perform the granule quantification, without fMLP treatment groups as control. Data represent the mean ± s.e.m. [(**p* < 0.05 *versus* the control group, NS means no different *versus* the not stimulated groups; Significance calculated using one-way ANOVA].

Supplementary Figure 4 Flow cytometry images of the surface expression of CD11b and CD18 on control and PA-treated dairy cow neutrophils.

Supplementary Figure 5 The viability of BSA control and PA (0.25 mM)-treated neutrophils was assessed using the CCK-8 cytotoxicity assay.

Supplementary Figure 6 Neutrophil lysosomal membrane permeabilization (LMP) of fatty liver neutrophils and PA-treated neutrophils was assessed using the acridine orange (AO) uptake assay. Data represent the mean ± s.e.m. (***p* < 0.01 versus the control group. Significance calculated using one-way ANOVA).

Supplementary Figure 7 Immunoblot for the total protein expression of CD11b and CD18 in control and PA-treated dHL-60 cells (infected with LV-GFP-shp62) was determined (*n* = 3). Data represent the mean ± s.e.m. (***p* < 0.01 versus the control group, ^##^ *p* < 0.01 and versus the PA-treated group; Significance calculated using two-way ANOVA).
