# Supplementary figures and images for: Autophagy Induced by Palmitic Acid Regulates Neutrophil Adhesion Through the Granule-Dependent Degradation of αMβ2 Integrin in Dairy Cows With Fatty Liver
Source: Front Immunol. 2021 Oct 7;12:726829. doi: 10.3389/fimmu.2021.726829 (PMC8529007; doi:10.3389/fimmu.2021.726829)

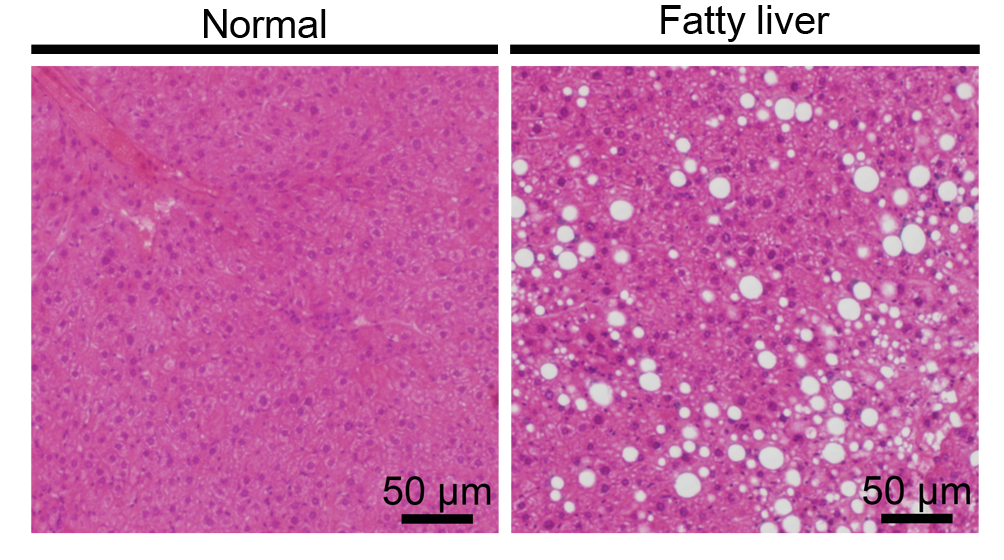

Supplement: Supplementary Figure 1 — Representative hematoxylin and eosin (H&E)-stained images of liver tissues in normal and fatty liver cows. [file Image_1.tif]

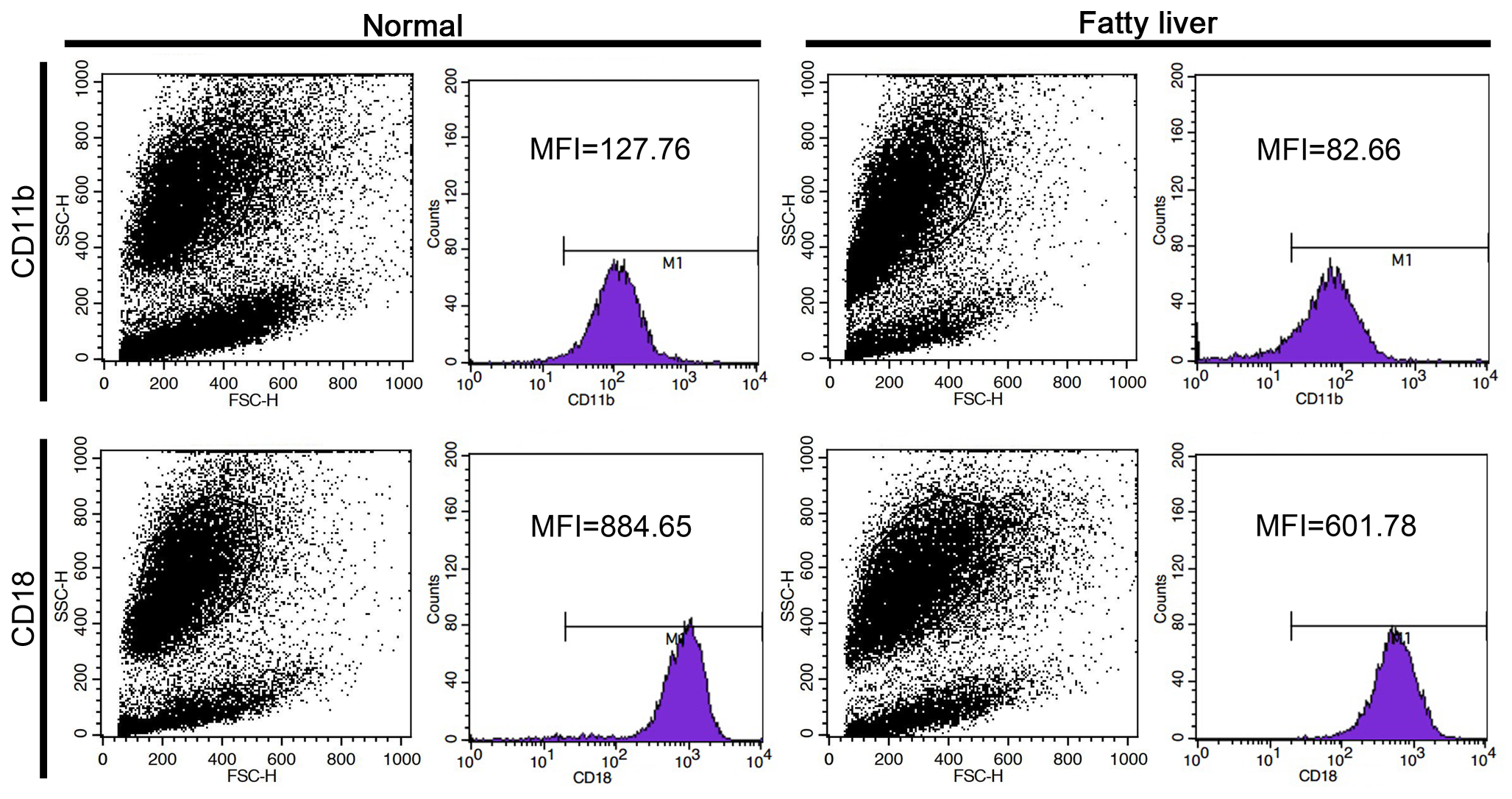

Supplement: Supplementary Figure 2 — Flow cytometry images of the surface expression of CD11b and CD18 on Normal and Fatty liver dairy cow neutrophils. [file Image_2.tif]

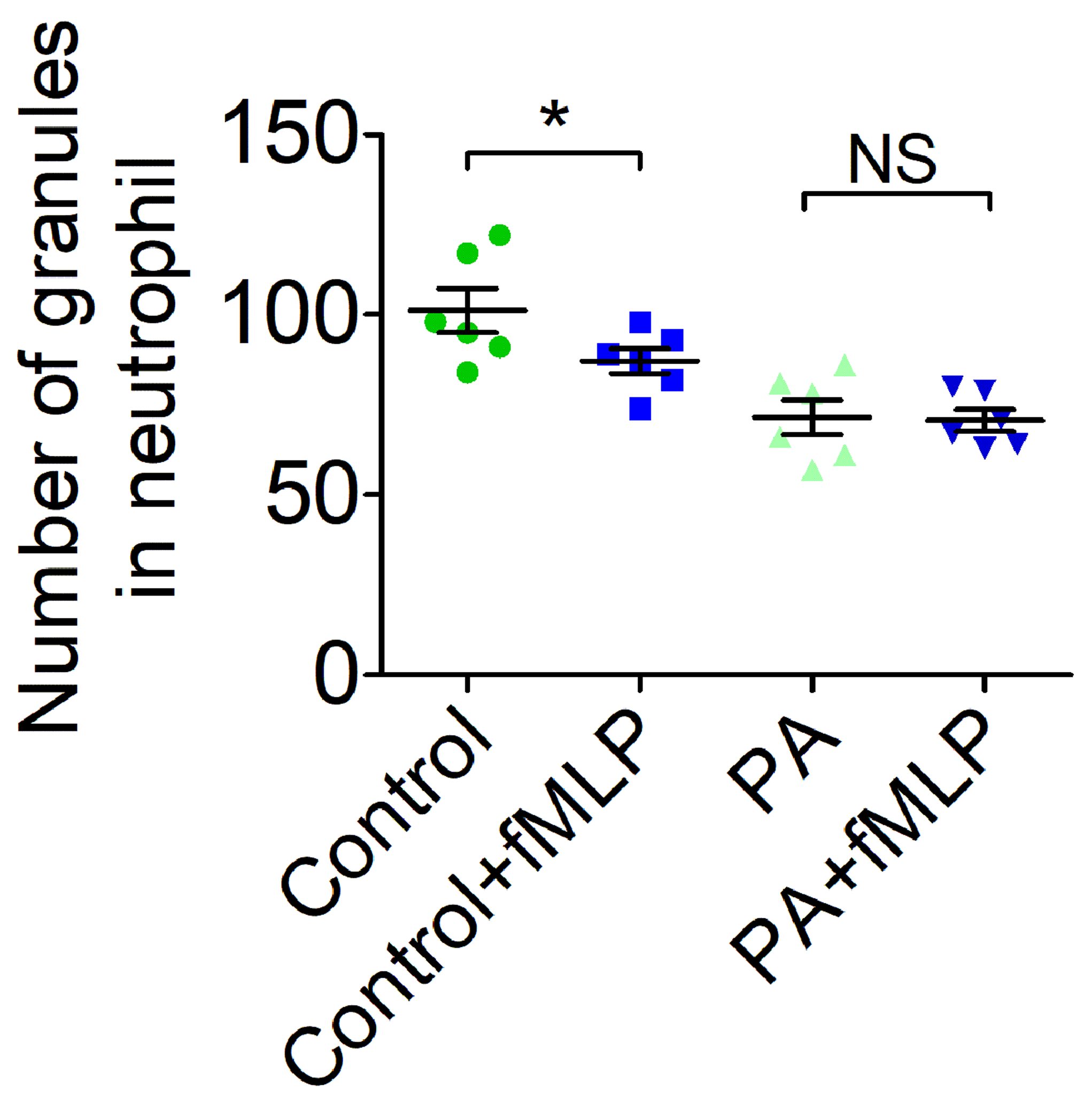

Supplement: Supplementary Figure 3 — The number of granules in normal and PA-treated neutrophils with or without using fMLP to induce degranulation. Control and PA-treated neutrophils were cultured for 6h and then stimulated with 1 μM fMLP for 30 min to induce degranulation and then were collected to perform the granule quantification, without fMLP treatment groups as control. Data represent the mean ± s.e.m. [(*p < 0.05 versus the control group, NS means no different versus the not stimulated groups; Significance calculated using one-way ANOVA]. [file Image_3.tif]

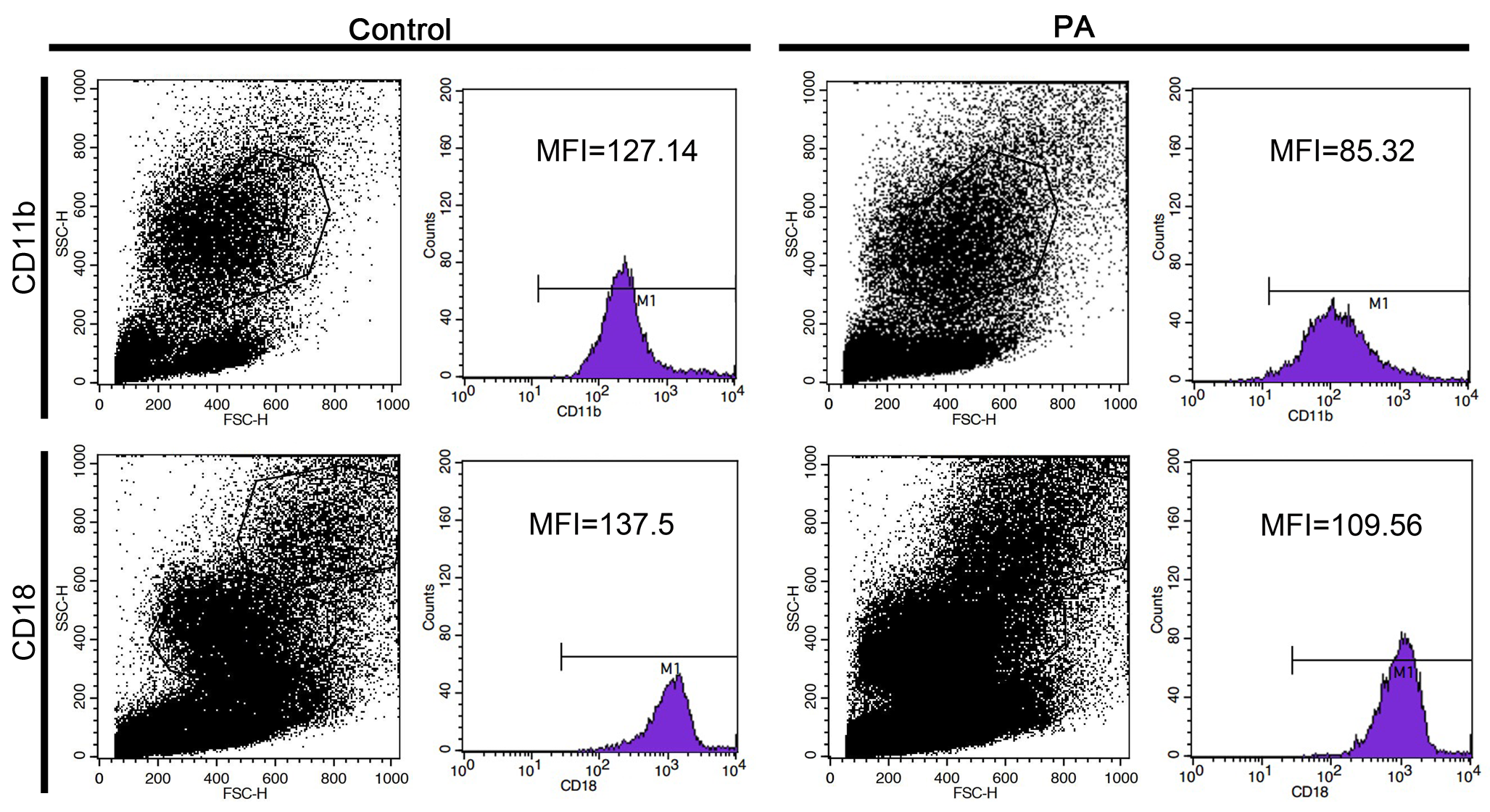

Supplement: Supplementary Figure 4 — Flow cytometry images of the surface expression of CD11b and CD18 on control and PA-treated dairy cow neutrophils. [file Image_4.tif]

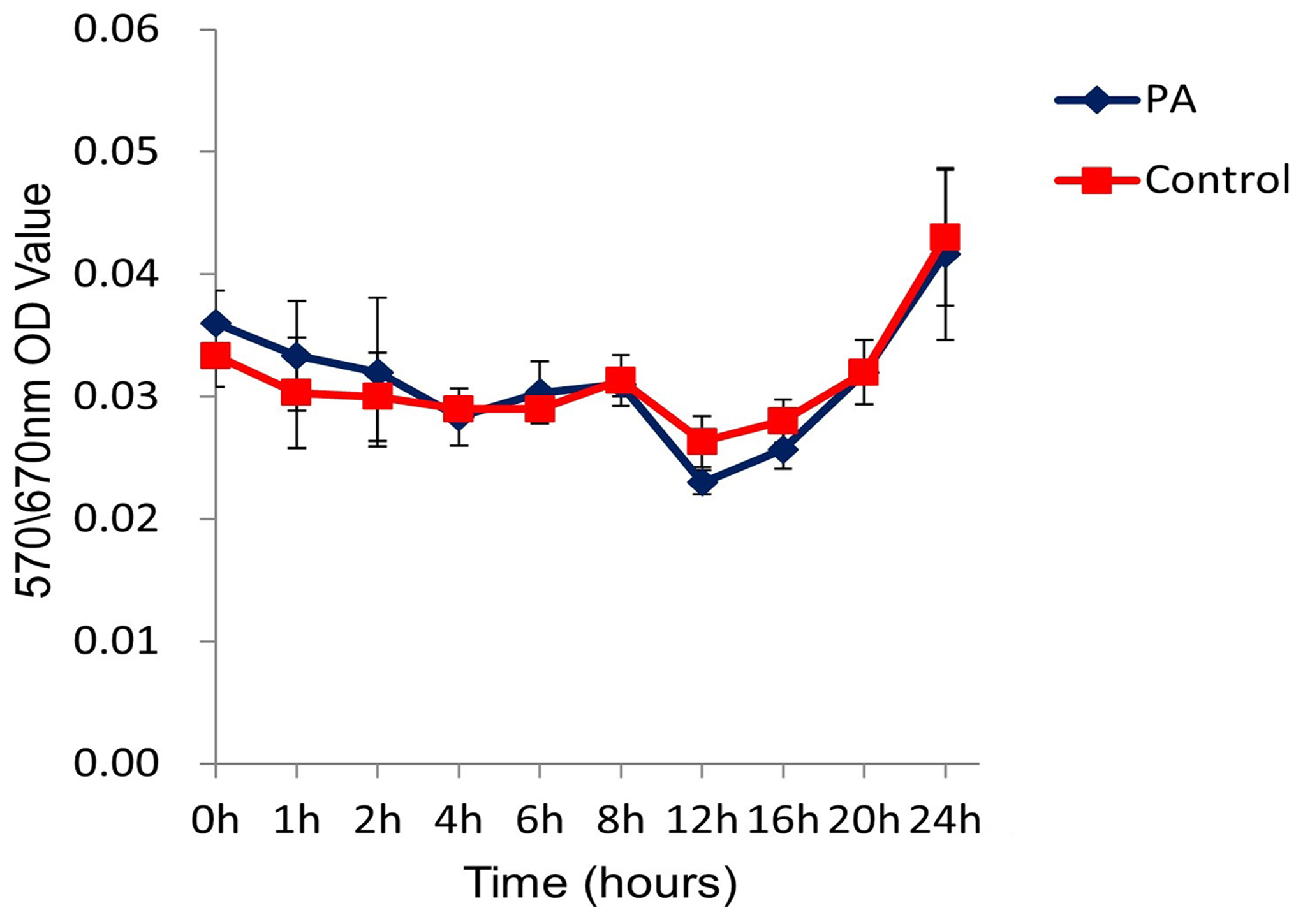

Supplement: Supplementary Figure 5 — The viability of BSA control and PA (0.25 mM)-treated neutrophils was assessed using the CCK-8 cytotoxicity assay. [file Image_5.tif]

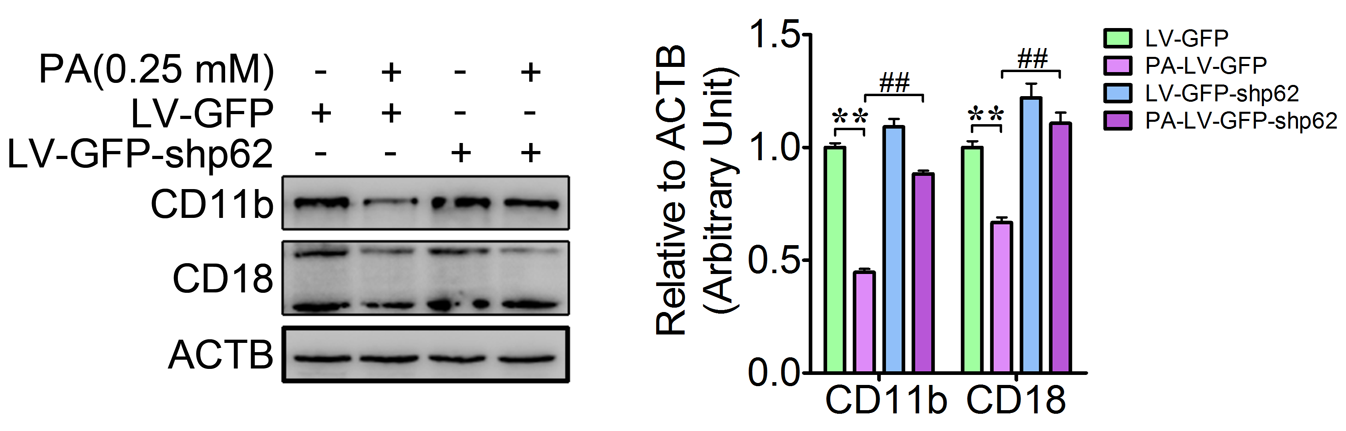

Supplement: Supplementary Figure 6 — Neutrophil lysosomal membrane permeabilization (LMP) of fatty liver neutrophils and PA-treated neutrophils was assessed using the acridine orange (AO) uptake assay. Data represent the mean ± s.e.m. (**p < 0.01 versus the control group. Significance calculated using one-way ANOVA). [file Image_6.tif]

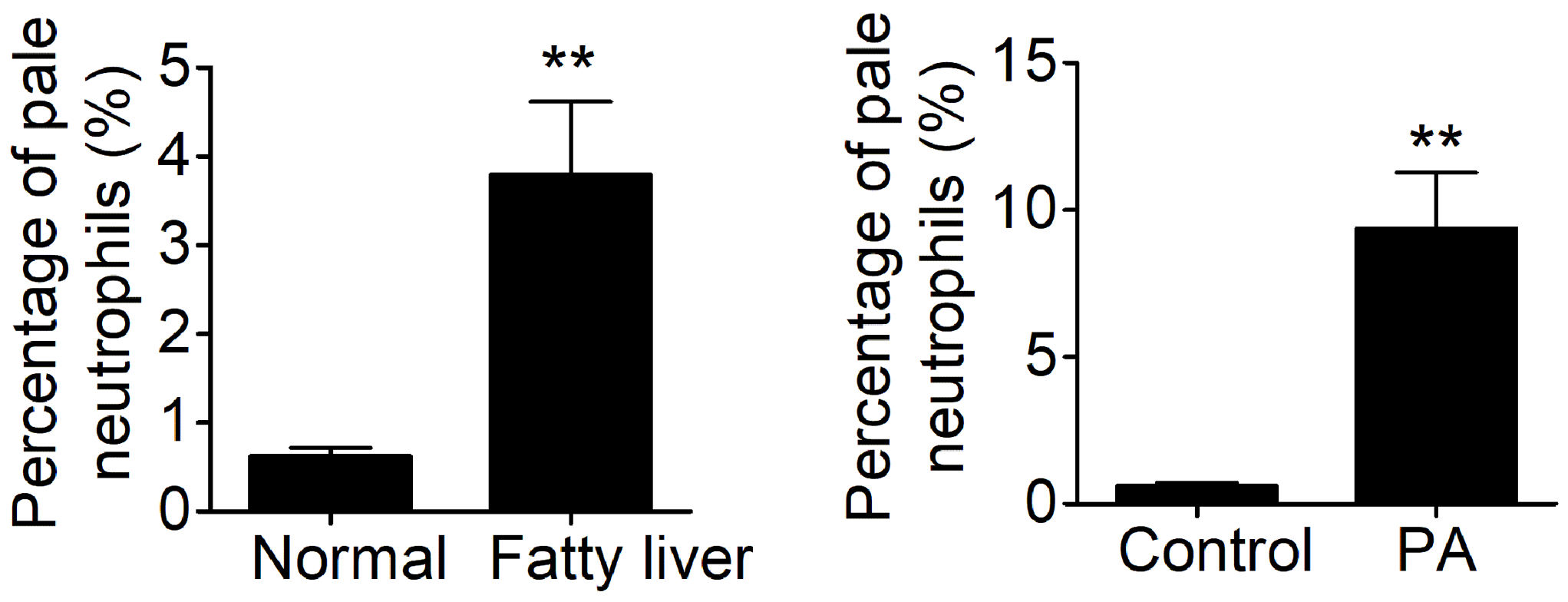

Supplement: Supplementary Figure 7 — Immunoblot for the total protein expression of CD11b and CD18 in control and PA-treated dHL-60 cells (infected with LV-GFP-shp62) was determined (n = 3). Data represent the mean ± s.e.m. (**p < 0.01 versus the control group, ## p < 0.01 and versus the PA-treated group; Significance calculated using two-way ANOVA). [file Image_7.tif]
