# Supplementary material for: Autophagy Induced by Palmitic Acid Regulates Neutrophil Adhesion Through the Granule-Dependent Degradation of αMβ2 Integrin in Dairy Cows With Fatty Liver
Source: Front Immunol. 2021 Oct 7;12:726829. doi: 10.3389/fimmu.2021.726829 (PMC8529007; doi:10.3389/fimmu.2021.726829)
Supplement: Supplementary file 8 [file Table_1.docx]

Table S1 The list of proteins interacting with CD11b identified by the shotgun

| Accession | Protein | Gene | Peptides | Unique Peptides | coverage | MW | PI |
| --- | --- | --- | --- | --- | --- | --- | --- |
| P32592 | Integrin beta-2 | ITGB2 | 88 | 37 | 48.89% | 84399.44 | 6.05 |
| G3MYD9 | Integrin alpha-M | ITGAM | 87 | 38 | 37.33% | 127652.6 | 7.13 |
| P24627 | Lactoferrin | LTF | 26 | 21 | 33.05% | 78055.53 | 8.69 |
| P22226 | Cathelicidin-1 | CATHL1 | 26 | 9 | 54.84% | 17599.87 | 7.58 |
| P54230 | Cathelicidin-1 | CATHL1A | 26 | 8 | 51.61% | 17647.98 | 7.54 |
| P19120 | Heat shock cognate 71 kDa protein | HSPA8 | 23 | 16 | 27.69% | 71239.66 | 5.37 |
| P63258 | Actin, cytoplasmic 2 | ACTG1 | 23 | 13 | 40.53% | 41792.4 | 5.31 |
| Q0VCX2 | 78 kDa glucose-regulated protein | HSPA5 | 22 | 19 | 36.18% | 72399.13 | 5.07 |
| Q3ZCH0 | Stress-70 protein, mitochondrial | HSPA9 | 19 | 18 | 29.01% | 73740.74 | 5.97 |
| E1BLI9 | S100-A9 | S100A9 | 15 | 4 | 33.33% | 16376.91 | 6.29 |
| W5NTG5 | Cathelicidin-1 | CATHL1B | 13 | 6 | 35.06% | 17574.95 | 8.6 |
| P2878 | S100-A8 | S100A8 | 13 | 5 | 40.45% | 10459.87 | 5.15 |
| Q27965 | Heat shock 70 kDa protein 1B | HSPA1B | 12 | 10 | 19.66% | 70227.56 | 5.68 |
| Q2HJI6 | Granulin | GRN | 12 | 9 | 19.76% | 63054.01 | 7.81 |
| P54228 | Cathelicidin-6 | CATHL6 | 12 | 8 | 46.84% | 17851.53 | 9.39 |
| P28783 | S100-A9 | S100A9 | 12 | 4 | 31.41% | 17113.66 | 6.29 |
| P02070 | Hemoglobin subunit beta | HBB | 11 | 7 | 57.24% | 15954.2 | 7.02 |
| P56425 | Cathelicidin-7 | CATHL7 | 11 | 6 | 34.55% | 18847.28 | 9.23 |
| P46171 | Beta-defensin 13 | DEFB13 | 11 | 4 | 88.10% | 4450.31 | 9.56 |
| A6QQA8 | Sulfhydryl oxidase | QSOX1 | 10 | 10 | 19.22% | 62974.43 | 9.26 |
| P79362 | Cathelicidin-2 | CATHL2 | 10 | 5 | 21.59% | 19841.6 | 9.6 |
| P54229 | Cathelicidin-5 | CATHL5 | 10 | 5 | 24.53% | 17616.11 | 8.37 |
| L8I022 | Filamin-A | M91_05428 | 9 | 9 | 4.16% | 280689.6 | 5.72 |
| P02672 | Fibrinogen alpha chain | FGA | 9 | 9 | 13.33% | 67011.38 | 6.73 |
| P01966 | Hemoglobin subunit alpha | HBA | 9 | 7 | 61.97% | 15184.18 | 8.07 |
| A4IF97 | Myosin regulatory light chain 12B | MYL12B | 9 | 7 | 47.37% | 19691.87 | 4.72 |

| Accession | Protein | Gene | Peptides | Unique Peptides | coverage | MW | PI |
| --- | --- | --- | --- | --- | --- | --- | --- |
| Q8SPP7 | Peptidoglycan recognition protein 1 | PGLYRP1 | 9 | 6 | 37.37% | 21062.81 | 9.59 |
| P02253 | Histone H1.2 | HIST1H1C | 9 | 6 | 26.76% | 21355.42 | 11.01 |
| W5NVS8 | Cathelin-related peptide SC5 | SC5 | 9 | 5 | 25.31% | 17903.35 | 8.73 |
| Q50JA5 | Neutrophil beta-defensin 8 | nbd8 | 9 | 3 | 38.71% | 6936.33 | 11.2 |
| Q5W5G9 | Neutrophil beta-defensin 7 (Fragment) | bnbd7 | 9 | 3 | 52.38% | 4820.75 | 11.2 |
| Q2TBU0 | Haptoglobin | HP | 8 | 7 | 19.45% | 44858.68 | 7.83 |
| G3X6K8 | Haptoglobin | HP | 8 | 7 | 19.45% | 44872.71 | 7.83 |
| P19660 | Cathelicidin-2 | CATHL2 | 8 | 5 | 22.73% | 20029.8 | 9.19 |
| P60661 | Myosin light polypeptide 6 | MYL6 | 8 | 5 | 34.44% | 16929.88 | 4.56 |
| P46169 | Beta-defensin 11 | DEFB11 | 8 | 3 | 43.33% | 6506.88 | 10.66 |
| P46161 | Beta-defensin 3 | DEFB3 | 8 | 3 | 38.60% | 6324.49 | 11.2 |
| P46160 | Beta-defensin 2 | DEFB2 | 8 | 3 | 55.00% | 4648.53 | 11.2 |
| L8HVH6 | Neutrophil gelatinase-associated lipocalin | M91_12706 | 7 | 7 | 41.21% | 22853.88 | 9.33 |
| Q5W5H6 | Beta-defensin 404 (Fragment) | defb404 | 7 | 3 | 52.38% | 4810.72 | 11.2 |
| P10096 | Glyceraldehyde-3-phosphate dehydrogenase | GAPDH | 6 | 6 | 20.12% | 35867.68 | 8.51 |
| P0CB32 | Heat shock 70 kDa protein 1-like | HSPA1L | 6 | 5 | 10.61% | 70388.24 | 5.89 |
| P79105 | S100-A12 | S100A12 | 6 | 5 | 42.39% | 10685.1 | 5.92 |
| Q7YRV4 | E3 ubiquitin-protein ligase TRIM21 | TRIM21 | 6 | 5 | 9.81% | 54030.26 | 6.17 |
| P33046 | Cathelicidin-4 | CATHL4 | 6 | 4 | 29.86% | 16478.66 | 6.29 |
| A6QPP9 | C-X-C motif chemokine | PF4 | 6 | 3 | 36.44% | 12608.71 | 9.3 |
| P46168 | Beta-defensin 10 | DEFB10 | 6 | 2 | 25.81% | 6928.37 | 10.74 |
| A8E4P3 | STOM protein | STOM | 5 | 5 | 16.90% | 31288.04 | 6.63 |
| L8HQG0 | Azurocidin | M91_15530 | 5 | 5 | 27.24% | 26504.05 | 11.14 |
| E1BKT9 | Desmoplakin protein | DSP | 5 | 5 | 1.52% | 332378.9 | 6.47 |
| Q1JPB0 | Leukocyte elastase inhibitor | SERPINB1 | 5 | 5 | 9.81% | 42235.24 | 5.7 |
| L8HXY5 | Histone H1.5 | M91_12990 | 5 | 4 | 17.26% | 22693.98 | 10.91 |
| A0A0A7NLY8 | Cathelicidin 4 | CATHL4 | 5 | 3 | 18.06% | 16287.47 | 6.28 |
| D6BMU8 | CR4 receptor subunit alphaX | alphaX | 5 | 3 | 2.76% | 127669.8 | 6.04 |
| F1MUD2 | Histone H2B | HIST1H2BM | 4 | 4 | 26.19% | 13934.02 | 10.31 |

Table S1 The list of proteins interacting with CD11b identified by the shotgun

| Accession | Protein | Gene | Peptides | Unique Peptides | coverage | MW | PI |
| --- | --- | --- | --- | --- | --- | --- | --- |
| Q8SPJ1 | Junction plakoglobin | JUP | 4 | 4 | 6.04% | 81819.96 | 5.75 |
| Q29443 | Serotransferrin | TF | 4 | 3 | 3.98% | 77752.38 | 6.75 |
| W5P0B2 | LDL Receptor Related Protein 2 | LRP2 | 4 | 1 | 0.11% | 521953.1 | 5.09 |
| P46164 | Beta-defensin 6 | DEFB6 | 3 | 3 | 64.29% | 4838.72 | 10.74 |
| F1MMU4 | H1 Histone Family Member X | H1FX | 3 | 3 | 14.02% | 22360.7 | 10.71 |
| A5D984 | Pyruvate kinase | PKM2 | 3 | 3 | 5.27% | 57948.27 | 7.96 |
| P68103 | Elongation factor 1-alpha 1 | EEF1A1 | 3 | 3 | 10.17% | 50140.28 | 9.1 |
| A5PJY7 | Ribonuclease A Family Member 2 | RNASE2 | 3 | 2 | 9.49% | 18091.03 | 9.94 |
| B0JYN9 | Ras-related C3 botulinum toxin substrate 2 | RAC2 | 2 | 2 | 9.38% | 21423.59 | 7.52 |
| F1MIW8 | Desmoglein-1 | DSG1 | 2 | 2 | 2.88% | 112327.9 | 4.88 |
| G3MXK8 | Proteinase 3 | PRTN3 | 2 | 2 | 6.88% | 26750.35 | 9.05 |
| F1MMQ7 | CD79b Molecule | CD79B | 2 | 2 | 7.42% | 25711.21 | 6 |
| L8HVW2 | CD177 antigen | M91_09270 | 2 | 2 | 5.68% | 46093.58 | 6.08 |
| P00829 | ATP synthase subunit beta, mitochondrial | ATP5B | 2 | 2 | 4.55% | 56282.84 | 5.15 |
| P40673 | High mobility group protein B2 | HMGB2 | 2 | 2 | 12.44% | 24033.48 | 7.62 |
| O02775 | Enteric beta-defensin | EBD | 2 | 2 | 28.13% | 7126.52 | 10.66 |
| P12763 | Alpha-2-HS-glycoprotein | AHSG | 2 | 2 | 7.52% | 38418.34 | 5.26 |
| P15497 | Apolipoprotein A-I | APOA1 | 2 | 2 | 6.79% | 30275.95 | 5.71 |
| Q32PD5 | 40S ribosomal protein S19 | RPS19 | 2 | 2 | 13.10% | 16060.31 | 10.31 |
| Q32PI4 | Complement factor I | CFI | 2 | 2 | 2.75% | 68931.89 | 8.07 |
| Q3T149 | Heat shock protein beta-1 | HSPB1 | 2 | 2 | 11.94% | 22392.77 | 5.98 |
| Q3T165 | Prohibitin | PHB | 2 | 2 | 7.72% | 29803.71 | 5.57 |
| Q5E9F7 | Cofilin-1 | CFL1 | 2 | 2 | 18.67% | 18518.35 | 8.16 |
| A7YWC6 | SUB1 Homolog, Transcriptional Regulator | SUB1 | 2 | 2 | 13.39% | 14369.14 | 9.6 |
| F1MYQ6 | Integrin alpha-L | ITGAL | 2 | 1 | 1.72% | 128654 | 5.28 |
| G1DG16 | Host cell factor C1 regulator 1 | HCFC1R1 | 2 | 1 | 4.38% | 15126.14 | 7.87 |
| G3N2D7 | Immunoglobulin Lambda Like Polypeptide 1 | IGLL1 | 2 | 1 | 12.93% | 12112.05 | 4.78 |
| E1BMQ6 | Calcium-transporting ATPase | ATP2A3 | 2 | 1 | 2.30% | 109320.1 | 5.29 |

Table S1 The list of proteins interacting with CD11b identified by the shotgun

| Accession | Protein | Gene | Peptides | Unique Peptides | coverage | MW | PI |
| --- | --- | --- | --- | --- | --- | --- | --- |
| Q2KJH7 | Aldehyde dehydrogenase 18 family, member A1 | ALDH18A1 | 2 | 1 | 1.01% | 87193.06 | 6.86 |
| Q3ZC81 | Similar to Protein C11orf33 | MGC127492 | 2 | 1 | 1.34% | 59428.66 | 8.73 |
| F1N184 | Neurexophilin And PC-Esterase Domain Family Member 4 | NXPE4 | 2 | 1 | 1.34% | 59370.62 | 8.82 |
| L8IV02 | Zinc finger protein 638 | M91_04547 | 2 | 1 | 0.41% | 219325.4 | 5.82 |
| P46159 | Beta-defensin 1 | DEFB1 | 2 | 1 | 42.11% | 4278.05 | 8.98 |
| Q32KV4 | Cytoplasmic dynein 2 light intermediate chain 1 | DYNC2LI1 | 2 | 1 | 1.42% | 39711.3 | 8.48 |
| W5P6Y6 | Adenylate Kinase 8 | AK8 | 2 | 1 | 1.88% | 54812.89 | 6.25 |
| Q2YDL7 | CMT1A duplicated region transcript 4 protein homolog | CDRT4 | 2 | 1 | 3.05% | 18511.31 | 9.66 |
| A6QQV4 | Methyltransferase Like 3 | METTL3 | 2 | 1 | 1.38% | 64446.17 | 5.98 |
| A0A0A0MPA2 | Histone H2A | HIST1H2AJ | 1 | 1 | 6.92% | 14121.33 | 10.9 |
| A4Z8Q1 | Lysozyme | LYZ | 1 | 1 | 6.08% | 16485.76 | 8.87 |
| Q547S4 | Pancreatic anionic trypsinogen | TRYP8 | 1 | 1 | 10.53% | 26294.34 | 4.61 |
| A6QQQ4 | Fc Fragment Of IgE Receptor Ia | FCER1A | 1 | 1 | 3.85% | 29793.36 | 9.61 |
| A8E651 | EWS RNA Binding Protein 1 | EWSR1 | 1 | 1 | 2.14% | 68292.34 | 9.37 |
| G3N269 | Fatty acid-binding protein, epidermal | FABP5 | 1 | 1 | 6.67% | 15238.37 | 8.58 |
| F1N301 | Ribosomal Protein L22 | RPL22 | 1 | 1 | 10.16% | 14758.76 | 9.21 |
| E1B7I1 | RNA-binding protein MEX3A (Fragment) | MEX3A | 1 | 1 | 1.45% | 50467.51 | 7.93 |
| Q0P579 | Microtubule Associated Serine/Threonine Kinase 3 (Fragment) | MAST3 | 1 | 1 | 9.60% | 13319.87 | 8.55 |
| P01030 | Complement C4 (Fragments) | C4 | 1 | 1 | 1.09% | 101550 | 6.15 |
| E1BIK4 | C-X-C motif chemokine | PPBP | 1 | 1 | 7.56% | 12912.28 | 9.39 |
| E1BIR8 | Filaggrin Family Member 2 | FLG2 | 1 | 1 | 0.35% | 297243.5 | 9.45 |
| E1BJ67 | Cyclin-L1 | CCNL1 | 1 | 1 | 1.90% | 59729.07 | 10.71 |
| E1BPS1 | Bromodomain and PHD finger-containing protein 1 | BRPF1 | 1 | 1 | 0.66% | 137360.2 | 8.16 |

Table S1 The list of proteins interacting with CD11b identified by the shotgun

| Accession | Protein | Gene | Peptides | Unique Peptides | coverage | MW | PI |
| --- | --- | --- | --- | --- | --- | --- | --- |
| Q3ZBB6 | AP-4 complex subunit sigma-1 | AP4S1 | 1 | 1 | 3.47% | 16990.55 | 5.07 |
| F1MD58 | Caspase-7 (Fragment) | CASP7 | 1 | 1 | 2.17% | 31451.44 | 8.71 |
| Q29RM3 | Receptor expression-enhancing protein 5 | REEP5 | 1 | 1 | 6.88% | 21416.68 | 8.27 |
| Q75WB5 | 5-oxoprolinase | OPLAH | 1 | 1 | 0.47% | 137225.7 | 6.11 |
| F1MJ20 | Rho GTPase-activating protein 28 (Fragment) | ARHGAP28 | 1 | 1 | 0.85% | 79360.88 | 8.9 |
| F1MV72 | Dynein Axonemal Heavy Chain 3 | DNAH3 | 1 | 1 | 0.86% | 81276.62 | 6.08 |
| F1N0S8 | Tumor suppressor candidate 1 protein (Fragment) | TUSC1 | 1 | 1 | 4.25% | 23017.23 | 10.87 |
| F1N160 | Pre-B Lymphocyte 1 | VPREB1 | 1 | 1 | 3.27% | 26730.04 | 9.12 |
| A6QQ70 | Microtubule-associated protein | MAP1S | 1 | 1 | 1.22% | 112471.1 | 6.2 |
| F1N2W4 | Putative Polycomb group protein ASXL2 (Fragment) | ASXL2 | 1 | 1 | 1.00% | 149829.3 | 8.87 |
| Q08E58 | Tubulin tyrosine ligase-like family, member 12 | TTLL12 | 1 | 1 | 1.07% | 75535.69 | 5.09 |
| G1DFP9 | Growth hormone receptor | GHR | 1 | 1 | 1.47% | 68407.01 | 4.61 |
| P35662 | Cylicin-1 | CYLC1 | 1 | 1 | 1.05% | 74816.41 | 9.76 |
| B0JYN2 | RAN, member RAS oncogene family | RAN | 1 | 1 | 4.63% | 24422.81 | 7.01 |
| O46606 | Phospholipase DDHD1 | DDHD1 | 1 | 1 | 2.17% | 97574.99 | 5.61 |
| E1BP92 | Vertnin | VRTN | 1 | 1 | 2.74% | 73598.91 | 9.83 |
| G5E6T2 | Rho GTPase Activating Protein 23 | ARHGAP23 | 1 | 1 | 0.76% | 157585.8 | 9.43 |
| E1BJK2 | Tubulin beta-1 chain | TUBB1 | 1 | 1 | 2.67% | 49986.9 | 4.99 |
| Q9XSI3 | 60S ribosomal protein L10 | RPL10 | 1 | 1 | 3.74% | 24602.65 | 10.16 |
| L8HPZ2 | Cadherin-4 (Fragment) | M91_09401 | 1 | 1 | 2.10% | 93880.21 | 4.74 |
| Q9XSA7 | Chloride intracellular channel protein 4 | CLIC4 | 1 | 1 | 2.37% | 28726.74 | 5.6 |
| F1N4L1 | Anion exchange protein (Fragment) | SLC4A2 | 1 | 1 | 0.48% | 137012.7 | 5.9 |
| Q0VCW4 | L-serine dehydratase/L-threonine deaminase | SDS | 1 | 1 | 3.36% | 34440.68 | 7 |
| F1MDL2 | Protein inturned | INTU | 1 | 1 | 0.96% | 104452.9 | 6.07 |
| F1MQQ1 | Ellis-van Creveld syndrome protein | EVC | 1 | 1 | 1.01% | 112251.9 | 6.15 |

Table S1 The list of proteins interacting with CD11b identified by the shotgun

| Accession | Protein | Gene | Peptides | Unique Peptides | coverage | MW | PI |
| --- | --- | --- | --- | --- | --- | --- | --- |
| E1BDF0 | Nexilin | NEXN | 1 | 1 | 2.07% | 80876.51 | 5.27 |
| E1BG12 | Peptidase inhibitor R3HDML (Fragment) | R3HDML | 1 | 1 | 4.78% | 28311.39 | 9.73 |
| E1B7R4 | Eukaryotic translation initiation factor 3 subunit A | EIF3A | 1 | 1 | 0.58% | 166274.8 | 6.33 |
| L8I4W0 | STE20-like serine/threonine-protein kinase | M91_18619 | 1 | 1 | 0.56% | 143028.9 | 5.02 |
| P68509 | 14-3-3 protein eta | YWHAH | 1 | 1 | 2.85% | 28211.42 | 4.81 |
| L8I6Q6 | Band 4.1-like protein 5 | M91_16211 | 1 | 1 | 1.78% | 49847.45 | 6.35 |
| W5PZH0 | UPF0577 protein KIAA1324-like protein | KIAA1324L | 1 | 1 | 0.72% | 106903.4 | 5.29 |
| Q76LV1 | Heat shock protein HSP 90-beta | HSP90AB1 | 1 | 1 | 1.66% | 83252.2 | 4.97 |
| F1N4V2 | RNA polymerase-associated protein CTR9-like protein | CTR9 | 1 | 1 | 1.19% | 134005.4 | 6.33 |
| E1BM52 | Forkhead box protein P4 | FOXP4 | 1 | 1 | 1.21% | 88806.14 | 6.63 |
| A0JN40 | Kinesin-like protein KIF3C | KIF3C | 1 | 1 | 0.76% | 89486.33 | 8.44 |
| P34955 | Alpha-1-antiproteinase | SERPINA1 | 1 | 1 | 2.64% | 46103.42 | 6.05 |
| P19483 | ATP synthase subunit alpha, mitochondrial | ATP5A1 | 1 | 1 | 1.81% | 59718.91 | 9.21 |
| L8IS94 | Leucine-rich repeat and IQ domain-containing protein 3 | M91_20040 | 1 | 1 | 1.62% | 72965.99 | 9.53 |
| E1BI01 | Dual specificity mitogen-activated protein kinase kinase 5 | MAP2K5 | 1 | 1 | 1.57% | 50106.13 | 6.03 |
| Q3MHM7 | 60S ribosomal protein L35 | RPL35 | 1 | 1 | 8.13% | 14565.32 | 11.04 |
| Q762I5 | Resistin | RETN | 1 | 1 | 12.84% | 11469.4 | 8 |
| Q08E36 | Transmembrane protein 198 | TMEM198 | 1 | 1 | 2.50% | 39608.4 | 9.97 |
| Q3SZ55 | Pentatricopeptide repeat-containing protein 2 | PTCD2 | 1 | 1 | 1.56% | 43944.85 | 9.05 |
| A7YWC4 | ATPase family AAA domain-containing protein 3 | ATAD3 | 1 | 1 | 1.19% | 66106.39 | 9.29 |
| Q28009 | RNA-binding protein FUS | FUS | 1 | 1 | 2.73% | 52310.26 | 9.4 |

Table S1 The list of proteins interacting with CD11b identified by the shotgun

| Accession | Protein | Gene | Peptides | Unique Peptides | coverage | MW | PI |
| --- | --- | --- | --- | --- | --- | --- | --- |
| A7MBC0 | Putative N-acetylglucosamine-6- phosphate deacetylase | AMDHD2 | 1 | 1 | 1.71% | 43495.02 | 5.93 |
| P10103 | High mobility group protein B1 | HMGB1 | 1 | 1 | 6.05% | 24907.51 | 5.62 |
| E1BMJ0 | Serpin Family G Member 1 | SERPING1 | 1 | 1 | 2.14% | 51710.64 | 6.2 |
| P79103 | 40S ribosomal protein S4 | RPS4 | 1 | 1 | 3.42% | 29597.37 | 10.16 |
| Q148F2 | Arachidonate 5-lipoxygenase-activating protein | ALOX5AP | 1 | 1 | 4.97% | 18027.83 | 8.58 |
| Q17QF4 | 5'-nucleotidase, cytosolic III-like | NT5C3L | 1 | 1 | 6.30% | 14926.22 | 9.24 |
| F1MLB9 | 5'-nucleotidase | NT5C3B | 1 | 1 | 2.74% | 33890.31 | 5.75 |
| Q28178 | Thrombospondin-1 | THBS1 | 1 | 1 | 0.94% | 129532.7 | 4.74 |
| Q29439 | Complement component C4 (Fragment) | C4 | 1 | 1 | 11.63% | 14474.08 | 5.33 |
| Q29RR7 | Vacuolar protein sorting-associated protein 37A | VPS37A | 1 | 1 | 3.02% | 44248.76 | 5.29 |
| Q2ABC0 | Taste receptor type 2 | T2R10C | 1 | 1 | 1.62% | 35221.13 | 9.64 |
| Q2KHX7 | RNA guanylyltransferase and 5'-phosphatase | RNGTT | 1 | 1 | 1.01% | 68377.73 | 8.34 |
| Q30DP7 | Type II small proline-rich protein (Fragment) | SPRR2A | 1 | 1 | 16.67% | 5942.02 | 8.39 |
| Q3SZ10 | 60S ribosomal protein L3-like | RPL3L | 1 | 1 | 1.97% | 46409.57 | 10.4 |
| Q3SZC1 | GrpE protein homolog 1, mitochondrial | GRPEL1 | 1 | 1 | 5.07% | 24305.73 | 8.26 |
| Q3T076 | Ribosomal protein S14 | RPS14 | 1 | 1 | 7.01% | 16776.28 | 10.04 |
| Q3ZC09 | Beta-enolase | ENO3 | 1 | 1 | 1.38% | 47095.46 | 7.6 |
| A6QR19 | ENO2 protein | ENO2 | 1 | 1 | 1.38% | 47268.03 | 4.94 |
| F1MB08 | Alpha-enolase | ENO1 | 1 | 1 | 1.38% | 47283.5 | 6.38 |
| Q3ZCL0 | Cysteine-rich secretory protein 2 | CRISP3 | 1 | 1 | 3.69% | 27452.98 | 8.64 |
| Q4PKH3 | H(+)/Cl(-) exchange transporter 7 | CLCN7 | 1 | 1 | 0.62% | 88830.21 | 7.97 |
| Q5H9M7 | Dynein heavy chain (Fragment) | Bv1 | 1 | 1 | 0.31% | 327819.8 | 6.13 |
| F1N724 | Dynein Axonemal Heavy Chain 11 | DNAH11 | 1 | 1 | 0.20% | 520795.6 | 5.82 |
| P63048 | Ubiquitin-60S ribosomal protein L40 | UBA52 | 1 | 1 | 12.50% | 14728.16 | 9.87 |
| P0CH28 | Polyubiquitin-C | UBC | 1 | 1 | 2.32% | 77569.24 | 7.16 |
| P0CG53 | Polyubiquitin-B | UBB | 1 | 1 | 5.25% | 34308.04 | 6.94 |
| Q7SIH1 | Alpha-2-macroglobulin | A2M | 1 | 1 | 0.73% | 167574.1 | 5.71 |
| U5N2I3 | Major prion protein (Fragment) | PRNP | 1 | 1 | 6.61% | 13492.95 | 8.69 |
| F1MKZ3 | Calcium-dependent secretion activator (Fragment) | CADPS | 1 | 1 | 1.16% | 137650.4 | 5.55 |

Table S1 The list of proteins interacting with CD11b identified by the shotgun

| Accession | Protein | Gene | Peptides | Unique Peptides | coverage | MW | PI |
| --- | --- | --- | --- | --- | --- | --- | --- |
| Q3SZX8 | Coiled-coil domain-containing protein 25 | CCDC25 | 1 | 1 | 4.33% | 24617.77 | 6.13 |
| F1MSP8 | Dynein Axonemal Heavy Chain 10 | DNAH10 | 1 | 1 | 0.22% | 514200.2 | 5.41 |
| W5NXM3 | NAD(P)(+)--arginine ADP-ribosyltransferase | LOC101118216 | 1 | 1 | 2.59% | 34590.02 | 7.69 |
| W5NY05 | Leucine, Glutamate And Lysine Rich 1 | LEKR1 | 1 | 1 | 1.01% | 81161.84 | 8.41 |
| A3KMY7 | PCTK3 protein | PCTK3 | 1 | 1 | 2.12% | 54125.54 | 9.26 |
| W5P2Y7 | THO Complex 5 | THOC5 | 1 | 1 | 1.10% | 72295.71 | 7.34 |
| A0JN35 | Ectodermal-neural cortex (With BTB-like domain) | ENC1 | 1 | 1 | 1.36% | 66101.08 | 6.4 |
| F1N592 | Kelch Like Family Member 25 | KLHL25 | 1 | 1 | 1.36% | 65776.19 | 6.27 |
| A6QP06 | Synaptotagmin-like protein 2 | SYTL2 | 1 | 1 | 0.64% | 105849.3 | 6.33 |
| W5P4G4 | RAB7B, Member RAS Oncogene Family | RAB7B | 1 | 1 | 3.00% | 22640.78 | 5.49 |
| F1MLM2 | Helicase-like transcription factor | HLTF | 1 | 1 | 0.79% | 113554.9 | 8.68 |
| W5P7K8 | BarH Like Homeobox 1 | BARHL1 | 1 | 1 | 1.83% | 35143.09 | 9.6 |
| Q28910 | Mucin (Fragment) | mucin | 1 | 1 | 1.00% | 63998.94 | 4.29 |
| F1MD73 | Deleted In Malignant Brain Tumors 1 | DMBT1 | 1 | 1 | 0.34% | 190097.3 | 5.52 |
| A5D792 | Histone H4 | DCK | 1 | 1 | 6.54% | 17680.22 | 9.95 |
| Q2TA49 | Vasodilator-stimulated phosphoprotein | VASP | 1 | 1 | 3.13% | 40462.72 | 8.78 |
| W5PTX3 | Secretory Carrier Membrane Protein 4 | SCAMP4 | 1 | 1 | 2.87% | 30671.53 | 9.37 |
| A8QQM6 | RAN Binding Protein 9 (Fragment) | RANBP9 | 1 | 1 | 4.58% | 26780.67 | 7.85 |
| Q56JX3 | 60S ribosomal protein L31 | RPL31 | 1 | 1 | 7.20% | 14462.69 | 10.54 |
| A2VE26 | Exostosin Like Glycosyltransferase 3 | EXTL3 | 1 | 1 | 0.65% | 104305.4 | 6.1 |
| F1MI97 | Actin-binding LIM protein 1 | ABLIM1 | 1 | 1 | 0.89% | 88864.81 | 9.01 |
| F1MNR2 | Paired box protein Pax-2 | PAX2 | 1 | 1 | 1.44% | 44705.37 | 7.25 |
| W5Q3B9 | Kelch Like Family Member 34 | KLHL34 | 1 | 1 | 1.81% | 48799.74 | 5.19 |
| F1N0A6 | Adhesion G Protein-Coupled Receptor V1 | ADGRV1 | 1 | 1 | 0.09% | 598953.9 | 4.48 |
| A6QQV8 | Lamin B Receptor | LBR | 1 | 1 | 1.61% | 71253.97 | 9.41 |
| F1MRI0 | Guanylate cyclase | GUCY2F | 1 | 1 | 0.54% | 124236.5 | 6.34 |
| A7YY47 | Lamin B1 | LMNB1 | 1 | 1 | 1.71% | 66420.45 | 5.08 |
| E1BK15 | Chloride channel protein | CLCN6 | 1 | 1 | 0.69% | 97233.48 | 6.52 |
| W5QII4 | Dmx Like 2 | DMXL2 | 1 | 1 | 0.23% | 342815.6 | 5.93 |
