# Supplementary material for: Autophagy Induced by Palmitic Acid Regulates Neutrophil Adhesion Through the Granule-Dependent Degradation of αMβ2 Integrin in Dairy Cows With Fatty Liver
Source: Front Immunol. 2021 Oct 7;12:726829. doi: 10.3389/fimmu.2021.726829 (PMC8529007; doi:10.3389/fimmu.2021.726829)
Supplement: Supplementary file 9 [file Table_2.docx]

Table S2 The list of proteins interacting with CD18 identified by the shotgun

| Accession | Protein | Gene | Peptides | Unique Peptides | coverage | MW | PI |
| --- | --- | --- | --- | --- | --- | --- | --- |
| G3MYD9 | Integrin alpha-M | ITGAM | 282 | 55 | 54.43% | 127652.6 | 7.13 |
| P32592 | Integrin beta-2 | ITGB2 | 233 | 42 | 54.36% | 84399.44 | 6.05 |
| Q2VJ42 | Integrin beta-2 | ITGB2 | 155 | 27 | 36.88% | 84487.56 | 6.32 |
| A2TJJ3 | Integrin alpha-M | ITGAM | 122 | 27 | 22.92% | 127549.7 | 7.32 |
| A2TJJ2 | Integrin alpha-M | ITGAM | 97 | 27 | 22.48% | 127602.8 | 7.31 |
| P61625 | Integrin alpha-L | ITGAL | 63 | 30 | 30.99% | 128724.1 | 5.26 |
| D6BMU8 | CR4 receptor subunit alphaX | alphaX | 61 | 14 | 12.78% | 127669.8 | 6.04 |
| P02672 | Fibrinogen alpha chain | FGA | 51 | 26 | 42.28% | 67011.38 | 6.73 |
| W5PH85 | Integrin alpha-X | ITGAX | 51 | 10 | 9.99% | 127747.2 | 6.49 |
| W5PHL7 | Integrin alpha-D | ITGAD | 37 | 4 | 3.62% | 126867.3 | 6 |
| P60712 | Actin, cytoplasmic 1 | ACTB | 27 | 15 | 48.27% | 41736.29 | 5.29 |
| P54230 | Cathelicidin-1 | CATHL1A | 20 | 8 | 51.61% | 17647.98 | 7.54 |
| B9VPZ5 | Lactoferrin | LF | 19 | 18 | 28.53% | 78055.53 | 8.69 |
| P19120 | Heat shock cognate 71 kDa protein | HSPA8 | 19 | 16 | 29.54% | 71239.66 | 5.37 |
| Q0VCX2 | 78 kDa glucose-regulated protein | HSPA5 | 17 | 15 | 27.79% | 72399.13 | 5.07 |
| Q3ZC07 | Actin, alpha cardiac muscle 1 | ACTC1 | 17 | 10 | 28.12% | 42018.52 | 5.23 |
| P68138 | Actin, alpha skeletal muscle | ACTA1 | 17 | 10 | 28.12% | 42050.59 | 5.23 |
| Q2HJI6 | Granulin | GRN | 16 | 10 | 21.47% | 63054.01 | 7.81 |
| Q9MZW1 | Beta-actin (Fragment) | ACTB | 16 | 9 | 50.44% | 25263.56 | 4.84 |
| A6QQA8 | Sulfhydryl oxidase | QSOX1 | 14 | 13 | 28.04% | 62974.43 | 9.26 |
| G3X6K8 | Haptoglobin | HP | 13 | 11 | 30.42% | 44872.71 | 7.83 |
| P28783 | S100-A9 | S100A9 | 13 | 4 | 31.41% | 17113.66 | 6.29 |
| W5NTG5 | Cathelicidin-1 | CATHL1B | 12 | 6 | 35.06% | 17574.95 | 8.6 |
| Q3ZCH0 | Stress-70 protein, mitochondrial | HSPA9 | 11 | 11 | 17.38% | 73740.74 | 5.97 |
| Q27965 | Heat shock 70 kDa protein 1B | HSPA1B | 11 | 10 | 16.85% | 70227.56 | 5.68 |
| Q27975 | Heat shock 70 kDa protein 1A | HSPA1A | 11 | 10 | 16.85% | 70257.65 | 5.68 |
| F1MHS5 | S100-A9 | S100A9 | 11 | 4 | 31.41% | 17097.62 | 6.29 |
| P01966 | Hemoglobin subunit alpha | HBA | 10 | 8 | 66.90% | 15184.18 | 8.07 |
| A6QQ97 | Integrin alpha-D | ITGAD | 10 | 7 | 6.78% | 127889.1 | 5.78 |
| P56425 | Cathelicidin-7 | CATHL7 | 10 | 7 | 39.39% | 18847.28 | 9.23 |
| P54228 | Cathelicidin-6 | CATHL6 | 10 | 7 | 39.24% | 17851.53 | 9.39 |
| P19660 | Cathelicidin-2 | CATHL2 | 10 | 6 | 26.14% | 20029.8 | 9.19 |
| A8E4P3 | STOM protein | STOM | 9 | 8 | 29.23% | 31288.04 | 6.63 |

Table S2 The list of proteins interacting with CD18 identified by the shotgun

| Accession | | Protein | | Gene | | Peptides | | Unique Peptides | | coverage | | MW | | | PI |
| --- | --- | --- | --- | --- | --- | --- | --- | --- | --- | --- | --- | --- | --- | --- | --- |
| E1B6Z6 | | neutrophil gelatinase-associated lipocalin (Fragment) | | LCN2 | | 9 | | 8 | | 43.00% | | 22982.99 | | | 9.35 |
| P33046 | | Cathelicidin-4 | | CATHL4 | | 9 | | 6 | | 35.42% | | 16478.66 | | | 6.29 |
| Q8SPP7 | | Peptidoglycan recognition protein 1 | | PGLYRP1 | | 9 | | 6 | | 39.47% | | 21062.81 | | | 9.59 |
| Q7YRV4 | | E3 ubiquitin-protein ligase TRIM21 | | TRIM21 | | 8 | | 8 | | 15.35% | | 54030.26 | | | 6.17 |
| L8I022 | | Filamin-A | | M91_05428 | | 8 | | 8 | | 3.74% | | 280689.6 | | | 5.72 |
| P02070 | | Hemoglobin subunit beta | | HBB | | 8 | | 7 | | 57.24% | | 15954.2 | | | 7.02 |
| P04346 | | Hemoglobin subunit beta-A | | HBB | | 8 | | 7 | | 56.55% | | 15964.15 | | | 6.36 |
| W5Q5X7 | | S100-A8 | | LOC101111791 | | 8 | | 6 | | 10.75% | | 50280.83 | | | 5.68 |
| P54229 | | Cathelicidin-5 | | CATHL5 | | 8 | | 5 | | 24.53% | | 17616.11 | | | 8.37 |
| P19661 | | Cathelicidin-3 | | CATHL3 | | 8 | | 5 | | 21.58% | | 21566.83 | | | 10.87 |
| P82018 | | Cathelicidin-2 | | CATHL2 | | 8 | | 5 | | 21.59% | | 19845.59 | | | 9.37 |
| P28782 | | S100-A8 | | S100A8 | | 7 | | 6 | | 60.67% | | 10459.87 | | | 5.15 |
| L8HQG0 | | Azurocidin | | M91_15530 | | 7 | | 6 | | 31.71% | | 26504.05 | | | 11.14 |
| P0CB32 | | Heat shock 70 kDa protein 1-like | | HSPA1L | | 7 | | 6 | | 9.98% | | 70388.24 | | | 5.89 |
| P50415 | | Cathelicidin-3 | | CATHL3 | | 7 | | 4 | | 14.21% | | 21829.21 | | | 10.9 |
| P79105 | | S100-A12 | | S100A12 | | 6 | | 5 | | 53.26% | | 10685.1 | | | 5.92 |
| P82017 | | MAP34-A protein | | map34-A | | 6 | | 5 | | 20.61% | | 18823.26 | | | 9.04 |
| W5Q6G0 | | LOC101111440 | | LOC101111440 | | 6 | | 5 | | 8.10% | | 57990.45 | | | 8.38 |
| W5Q7G1 | | LOC101118712 | | LOC101118712 | | 6 | | 5 | | 18.72% | | 24967.43 | | | 4.82 |
| P02662 | | Alpha-S1-casein | | CSN1S1 | | 6 | | 3 | | 17.29% | | 24528.64 | | | 4.98 |
| P46165 | | Beta-defensin 7 | | DEFB7 | | 6 | | 3 | | 35.48% | | 6964.39 | | | 11.47 |
| Q71UA5 | | Neutrophil beta-defensin 12 (Fragment) | | NBD12 | | 6 | | 2 | | 25.00% | | 6463.85 | | | 9.99 |
| Q28178 | | Thrombospondin-1 | | THBS1 | | 5 | | 5 | | 4.10% | | 129532.7 | | | 4.74 |
| Q1JPB0 | | Leukocyte elastase inhibitor | | SERPINB1 | | 5 | | 5 | | 11.41% | | 42235.24 | | | 5.7 |
| P10096 | | Glyceraldehyde-3-phosphate dehydrogenase | | GAPDH | | 5 | | 5 | | 15.62% | | 35867.68 | | | 8.51 |
| A0A0A7NLY8 | | Cathelicidin 4 | | CATHL4 | | 5 | | 4 | | 23.61% | | 16287.47 | | | 6.28 |
| P02253 | | Histone H1.2 | | HIST1H1C | | 5 | | 4 | | 19.25% | | 21355.42 | | | 11.01 |
| P46168 | | Beta-defensin 10 | | DEFB10 | | 5 | | 3 | | 40.32% | | 6928.37 | | | 10.74 |
| P46166 | | Beta-defensin 8 | | DEFB8 | | 5 | | 3 | | 63.16% | | 4359.24 | | | 10.66 |
| P46161 | | Beta-defensin 3 (Fragment) | | DEFB3 | | 5 | | 2 | | 28.07% | | 6324.49 | | | 11.2 |
| P46160 | | Beta-defensin 2 | | DEFB2 | | 5 | | 2 | | 40.00% | | 4648.53 | | | 11.2 |
| Accession | | Protein | | Gene | | Peptides | | Unique Peptides | | coverage | | MW | | PI |  |
| Q32KV4 | | Cytoplasmic dynein 2 light intermediate chain 1 | | DYNC2LI1 | | 5 | | 1 | | 1.42% | | 39711.3 | | 8.48 |  |
| P61223 | | Ras-related protein Rap-1b | | RAP1B | | 4 | | 4 | | 19.02% | | 20824.56 | | 5.65 |  |
| Q9TU25 | | Ras-related C3 botulinum toxin substrate 2 | | RAC2 | | 4 | | 4 | | 24.48% | | 21423.59 | | 7.52 |  |
| P46193 | | Annexin A1 | | ANXA1 | | 4 | | 4 | | 16.47% | | 38951.23 | | 6.38 |  |
| A5D984 | | Pyruvate kinase | | PKM2 | | 4 | | 4 | | 7.72% | | 57948.27 | | 7.96 |  |
| E1BJV5 | | Zinc finger protein 638 | | ZNF638 | | 4 | | 2 | | 0.66% | | 219062.4 | | 6.08 |  |
| P46169 | | Beta-defensin 11 | | DEFB11 | | 4 | | 2 | | 25.00% | | 6506.88 | | 10.66 |  |
| Q32S29 | | Histone H2B | | H2B | | 4 | | 2 | | 15.87% | | 13926.09 | | 10.27 |  |
| A0A0A7NSG7 | | Cathelicidin 4 | | CATHL4 | | 3 | | 3 | | 14.86% | | 17173.31 | | 5.57 |  |
| E1BJK2 | | Tubulin beta-1 chain | | TUBB1 | | 3 | | 3 | | 7.56% | | 49986.9 | | 4.99 |  |
| P19483 | | ATP synthase subunit alpha, mitochondrial | | ATP5A1 | | 3 | | 3 | | 6.15% | | 59718.91 | | 9.21 |  |
| P0CH28 | | Polyubiquitin-C | | UBC | | 3 | | 3 | | 3.04% | | 77569.24 | | 7.16 |  |
| P63048 | | Ubiquitin-60S ribosomal protein L40 | | UBA52 | | 3 | | 3 | | 16.41% | | 14728.16 | | 9.87 |  |
| P0CG53 | | Polyubiquitin-B | | UBB | | 3 | | 3 | | 6.89% | | 34308.04 | | 6.94 |  |
| P62992 | | Ubiquitin-40S ribosomal protein S27a | | RPS27A | | 3 | | 3 | | 13.46% | | 17964.72 | | 9.68 |  |
| L8HQA9 | | Cathelicidin-3 (Fragment) | | M91_03175 | | 3 | | 3 | | 17.24% | | 12881.59 | | 5.85 |  |
| L8HVW2 | | CD177 antigen | | M91_09270 | | 3 | | 3 | | 7.05% | | 46093.58 | | 6.08 |  |
| L8I0P2 | | Cathepsin G (Fragment) | | M91_02038 | | 3 | | 3 | | 14.29% | | 27989.06 | | 10.05 |  |
| Q2HJ86 | | Tubulin alpha-1D chain | | TUBA1D | | 3 | | 3 | | 9.07% | | 50282.23 | | 4.91 |  |
| Q32KN8 | | Tubulin alpha-3 chain | | TUBA3 | | 3 | | 3 | | 9.11% | | 49924.97 | | 4.98 |  |
| A4IF97 | | Myosin regulatory light chain 12B | | MYL12B | | 3 | | 3 | | 18.71% | | 19691.87 | | 4.72 |  |
| Q5E9E2 | | Myosin regulatory light  polypeptide 9 | | MYL9 | | 3 | | 3 | | 18.60% | | 19865 | | 4.67 |  |
| Q92176 | | Coronin-1A | | CORO1A | | 3 | | 3 | | 7.81% | | 50978.63 | | 6.25 |  |
| F1MD83 | | C-X-C motif chemokine | | PF4 | | 3 | | 2 | | 27.12% | | 12566.63 | | 9.3 |  |
| P67975 | | Beta-lactoglobulin | | LGB | | 3 | | 2 | | 10.49% | | 18150.92 | | 5.26 |  |
| W5PP29 | | Zinc finger protein 638 | | ZNF638 | | 3 | | 2 | | 0.56% | | 220024.2 | | 5.78 |  |
| F1MEQ7 | | Erythropoietin receptor | | EPOR | | 3 | | 1 | | 0.98% | | 55066.94 | | 4.79 |  |
| G1DG16 | | Host cell factor C1 regulator 1 | | HCFC1R1 | | 3 | | 1 | | 4.38% | | 15126.14 | | 7.87 |  |

Table S2 The list of proteins interacting with CD18 identified by the shotgun

| Accession | Protein | Gene | Peptides | Unique Peptides | coverage | MW | PI |
| --- | --- | --- | --- | --- | --- | --- | --- |
| G3N2D7 | Immunoglobulin Lambda Like Polypeptide 1 | IGLL1 | 3 | 1 | 12.93% | 12112.05 | 4.78 |
| A4IFU5 | Histone H2A | HIST3H2A | 2 | 2 | 10.77% | 14121.29 | 11.05 |
| Q2UVX4 | Complement C3 | C3 | 2 | 2 | 1.75% | 187251 | 6.41 |
| A7Z066 | Canx protein | canx | 2 | 2 | 3.37% | 67774.65 | 4.48 |
| Q5E9F7 | Cofilin-1 | CFL1 | 2 | 2 | 18.67% | 18518.35 | 8.16 |
| Q3SZI4 | 14-3-3 protein theta | YWHAQ | 2 | 2 | 8.57% | 27763.94 | 4.68 |
| F1MD73 | Deleted In Malignant Brain  Tumors 1 | DMBT1 | 2 | 2 | 1.20% | 190097.3 | 5.52 |
| F1MPV3 | RalA Binding Protein 1 | RALBP1 | 2 | 2 | 1.73% | 73717.77 | 5.7 |
| A6QQC9 | Olfactomedin 4 (Fragment) | OLFM4 | 2 | 2 | 5.08% | 49737.14 | 7.01 |
| Q32PI4 | Complement factor I | CFI | 2 | 2 | 2.91% | 68931.89 | 8.07 |
| G3MXK8 | Proteinase 3 | PRTN3 | 2 | 2 | 6.88% | 26750.35 | 9.05 |
| K4JB97 | Alpha-2-macroglobulin variant 4 | A2M | 2 | 2 | 6.01% | 42332.02 | 6.67 |
| Q5E9I6 | ADP-ribosylation factor | ARF3 | 2 | 2 | 10.50% | 20600.52 | 6.84 |
| P84080 | ADP-ribosylation factor 1 | ARF1 | 2 | 2 | 10.50% | 20696.53 | 6.31 |
| P84081 | ADP-ribosylation factor 2 | ARF2 | 2 | 2 | 10.50% | 20745.57 | 6.16 |
| P02663 | Alpha-S2-casein | CSN1S2 | 2 | 2 | 7.66% | 26018.39 | 8.55 |
| F1MDH3 | Talin-1 (Fragment) | TLN1 | 2 | 2 | 1.02% | 270812.4 | 5.81 |
| L8IE16 | Alpha-2-macroglobulin (Fragment) | M91_02447 | 2 | 2 | 1.06% | 167076.6 | 5.78 |
| P40673 | High mobility group protein B2 | HMGB2 | 2 | 2 | 12.44% | 24033.48 | 7.62 |
| Q9XSJ4 | Alpha-enolase | ENO1 | 2 | 2 | 4.38% | 47325.58 | 6.38 |
| Q1RMI2 | Ras homolog gene family, member G (Rho G) | RHOG | 2 | 2 | 10.47% | 21280.21 | 8.41 |
| P02584 | Profilin-1 | PFN1 | 2 | 2 | 20.00% | 15057.2 | 8.46 |
| Q3ZCL0 | Cysteine-rich secretory protein 2 | CRISP3 | 2 | 2 | 7.38% | 27452.98 | 8.64 |
| Q7SIH1 | Alpha-2-macroglobulin | A2M | 2 | 2 | 1.26% | 167574.1 | 5.71 |
| F1N0R5 | von Willebrand factor | VWF | 2 | 2 | 0.93% | 307677.1 | 5.39 |
| F1MM57 | Myosin-14 | MYH14 | 2 | 2 | 0.59% | 232361.9 | 5.58 |
| A6QPZ4 | SERPINB4 protein | SERPINB4 | 2 | 2 | 5.90% | 38646.75 | 6.19 |
| F1MEA6 | DIS3-like exonuclease 1 | DIS3L | 2 | 1 | 0.47% | 120576.5 | 6.04 |
| F1MVW3 | Integrin beta 8 | ITGB8 | 2 | 1 | 0.65% | 85583.34 | 7.53 |
| G3N250 | F-box only protein 48 | FBXO48 | 2 | 1 | 3.23% | 18246.61 | 7.63 |
| A1L580 | Semaphorin 6B | SEMA6B | 2 | 1 | 1.36% | 40499.38 | 9.15 |

Table S2 The list of proteins interacting with CD18 identified by the shotgun

| Accession | Protein | Gene | Peptides | Unique Peptides | coverage | MW | PI |
| --- | --- | --- | --- | --- | --- | --- | --- |
| E1BIT8 | Semaphorin-6B | SEMA6B | 2 | 1 | 0.64% | 84597.62 | 8.44 |
| Q148L3 | Dopey Family Member 1 (Fragment) | DOPEY1 | 2 | 1 | 0.77% | 73709.38 | 7.68 |
| E1BC71 | Doublesex-and mab-3-related transcription factor 2 | DMRT2 | 2 | 1 | 1.08% | 61379.55 | 6.53 |
| E1BEN3 | Kinesin-like protein | KIF17 | 2 | 1 | 0.68% | 115145.8 | 5.33 |
| Q3B7M7 | Unconventional prefoldin RPB5 interactor | URI1 | 2 | 1 | 1.15% | 58992.97 | 5.19 |
| F1N0A6 | G protein-coupled receptor 98 | ADGRV1 | 2 | 1 | 0.09% | 598953.9 | 4.48 |
| E1BLG8 | AMP deaminase 2 | AMPD2 | 2 | 1 | 0.88% | 92124.88 | 5.9 |
| W5P0B2 | LDL Receptor Related Protein 2 | LRP2 | 2 | 1 | 0.11% | 521953.1 | 5.09 |
| W5PW43 | URB1 Ribosome Biogenesis 1 Homolog | URB1 | 2 | 1 | 0.32% | 243856.5 | 8.05 |
| Q5E9B1 | L-lactate dehydrogenase B chain | LDHB | 1 | 1 | 3.29% | 36723.23 | 6.02 |
| P04272 | Annexin A2 | ANXA2 | 1 | 1 | 2.95% | 38611.63 | 6.92 |
| Q3MIB9 | TGFB Induced Factor Homeobox 1 | TGIF1 | 1 | 1 | 1.98% | 27577.95 | 8.61 |
| O46521 | Cytochrome b-245 light chain | CYBA | 1 | 1 | 5.24% | 20495.65 | 9.35 |
| A6QPB3 | Collagen alpha-1(XVII) chain | COL17A1 | 1 | 1 | 0.34% | 149152.1 | 8.82 |
| A6QPP7 | Neutrophil elastase | ELA2 | 1 | 1 | 4.87% | 28947.12 | 11.32 |
| A6QQJ0 | Spectrin Repeat Containing Nuclear Envelope Protein 1 | SYNE1 | 1 | 1 | 1.06% | 107336.7 | 5.22 |
| E1B8D0 | Lamin B Receptor | LBR | 1 | 1 | 1.61% | 71253.97 | 9.41 |
| E1B8D0 | Flap endonuclease GEN-like protein 1 | GEN1 | 1 | 1 | 0.88% | 103264.9 | 8.51 |
| E1B8Z0 | Bromodomain and PHD finger-containing protein 3 | BRPF3 | 1 | 1 | 0.68% | 132926.2 | 6.55 |
| E1BPS1 | Bromodomain and PHD finger-containing protein 1 | BRPF1 | 1 | 1 | 0.66% | 137360.2 | 8.16 |
| A5D792 | Histone H4 | DCK | 1 | 1 | 5.23% | 17680.22 | 9.95 |
| E1BLC2 | Histone H4 | HIST1H4I | 1 | 1 | 7.77% | 11381.23 | 11.36 |
| E1BJL9 | Coiled-Coil Domain  Containing 173 | CCDC173 | 1 | 1 | 0.90% | 65842.07 | 9.01 |
| F1MYX8 | Breast cancer type 1 susceptibility protein | BRCA1 | 1 | 1 | 0.27% | 206300.3 | 5.37 |

| Accession | Protein | Gene | Peptides | Unique Peptides | coverage | MW | PI |
| --- | --- | --- | --- | --- | --- | --- | --- |
| O18883 | Thioredoxin domain-containing protein 9 | TXNDC9 | 1 | 1 | 2.21% | 26503.84 | 5.86 |
| E1BF90 | Bestrophin-3 | BEST3 | 1 | 1 | 1.05% | 75821.53 | 8.35 |
| E1BGJ0 | LDL Receptor Related Protein 1 | LRP1 | 1 | 1 | 0.13% | 504755.8 | 5.13 |
| A7E306 | Nidogen 2 | NID2 | 1 | 1 | 0.46% | 142786.8 | 5.13 |
| E1BI01 | Dual specificity mitogen-activated protein kinase kinase 5 | MAP2K | 1 | 1 | 1.57% | 50106.13 | 6.03 |
| E1BKQ6 | Myosin XVIIIA | MYO18A | 1 | 1 | 0.29% | 232837.2 | 6.08 |
| E1BNR1 | Kelch-like protein 11 | KLHL11 | 1 | 1 | 3.57% | 20016.97 | 6.39 |
| E1BP31 | Ankyrin Repeat Domain 42 | ANKRD42 | 1 | 1 | 0.64% | 87163.41 | 6.15 |
| A7MB84 | Glypican 5 | GPC5 | 1 | 1 | 0.84% | 66354.24 | 8.3 |
| Q28156 | cGMP-specific 3',5'-cyclic phosphodiesterase | PDE5A | 1 | 1 | 0.58% | 98625.53 | 5.64 |
| E1BNY6 | Solute Carrier Family 35  Member D3 | SLC35D3 | 1 | 1 | 1.18% | 44850.64 | 6.95 |
| Q2KIT1 | PRKR-interacting protein 1 | PRKRIP1 | 1 | 1 | 6.45% | 21261.9 | 9.54 |
| Q75WB5 | 5-oxoprolinase | OPLAH | 1 | 1 | 0.47% | 137225.7 | 6.11 |
| Q3B7M3 | Family With Sequence Similarity 65 Member B | FAM65B | 1 | 1 | 0.79% | 111932.2 | 5.18 |
| Q3MHV6 | SLAIN motif-containing protein 2 | SLAIN2 | 1 | 1 | 0.86% | 62768.91 | 9.37 |
| M5FKI9 | KIAA1552 protein-like | FLYWCH1 | 1 | 1 | 0.99% | 78430.74 | 8.73 |
| F1MUS0 | Ran-binding protein 9 (Fragment) | RANBP9 | 1 | 1 | 2.04% | 58907.3 | 6.16 |
| Q3B7M0 | Zinc finger protein 207 | ZNF207 | 1 | 1 | 1.67% | 45185.84 | 8.99 |
| F1MXR8 | Zinc finger CCCH domain-containing protein 3 | ZC3H3 | 1 | 1 | 1.38% | 101378.4 | 10.91 |
| F1N160 | Pre-B Lymphocyte 1 | VPREB1 | 1 | 1 | 3.27% | 26730.04 | 9.12 |
| F1N371 | DAB2, Clathrin Adaptor Protein | DAB2 | 1 | 1 | 1.04% | 82465.22 | 5.57 |
| G3MYU7 | Tripartite Motif Containing 67 | TRIM67 | 1 | 1 | 0.71% | 92340.27 | 7.63 |
| O46783 | MHC class I heavy chain (Fragment) | MHC -I | 1 | 1 | 2.99% | 19648.47 | 5.21 |
| G5E618 | SH3 And Multiple Ankyrin Repeat Domains 1 | SHANK1 | 1 | 1 | 0.24% | 217434.1 | 8.05 |
| Q1LZC8 | WD repeat domain 13 | WDR13 | 1 | 1 | 2.31% | 23588.9 | 9.45 |

Table S2 The list of proteins interacting with CD18 identified by the shotgun

Table S2 The list of proteins interacting with CD18 identified by the shotgun

| Accession | Protein | Gene | Peptides | Unique Peptides | coverage | MW | PI |
| --- | --- | --- | --- | --- | --- | --- | --- |
| G5E608 | Protein Prenyltransferase Alpha Subunit Repeat Containing 1 | PTAR1 | 1 | 1 | 1.16% | 49553.13 | 6.58 |
| E1BJI7 | Ectopic P-Granules Autophagy Protein 5 Homolog | EPG5 | 1 | 1 | 0.20% | 290504.9 | 5.87 |
| F2FB42 | Mucin-5B | MUC5B | 1 | 1 | 0.07% | 689870.9 | 5.74 |
| F1MW16 | G Protein-Coupled Receptor 137C | GPR137C | 1 | 1 | 1.19% | 46419.35 | 7.55 |
| Q3ZC81 | Similar to Protein C11orf33 | MGC127492 | 1 | 1 | 1.34% | 59428.66 | 8.73 |
| F1MBK4 | Neurexophilin And PC-Esterase Domain Family Member 2 | NXPE2 | 1 | 1 | 1.33% | 60410.95 | 9.12 |
| F1N184 | Neurexophilin And PC-Esterase Domain Family Member 4 | NXPE4 | 1 | 1 | 1.34% | 59370.62 | 8.82 |
| E1BN71 | Guanylate cyclase | NPR1 | 1 | 1 | 0.85% | 119035 | 6.31 |
| F1MHT3 | Myosin VA | MYO5A | 1 | 1 | 0.86% | 216073.1 | 8.68 |
| Q0P5L6 | Centromere/kinetochore protein zw10 | ZW10 | 1 | 1 | 1.28% | 88416.02 | 5.74 |
| F1MYZ3 | Lysine Methyltransferase 2C | KMT2C | 1 | 1 | 0.15% | 526496.7 | 6.13 |
| L8HXY5 | Histone H1.5 | M91_12990 | 1 | 1 | 5.31% | 22693.98 | 10.91 |
| Q0VCW4 | L-serine dehydratase/L-threonine deaminase | SDS | 1 | 1 | 3.36% | 34440.68 | 7 |
| E1BM66 | Histone-lysine N-methyltransferase | NSD1 | 1 | 1 | 0.37% | 296637.2 | 8.38 |
| Q3SYV4 | Adenylyl cyclase-associated  protein 1 | CAP1 | 1 | 1 | 4.24% | 51272.06 | 7.16 |
| P60661 | Myosin light polypeptide 6 | MYL6 | 1 | 1 | 5.96% | 16929.88 | 4.56 |
| A7MB64 | Inositol 1,4,5-trisphosphate receptor-interacting protein | ITPRIP | 1 | 1 | 1.26% | 63431.13 | 5.71 |
| Q0P5E9 | HLA-B associated transcript 4 | BAT4 | 1 | 1 | 1.99% | 38581.88 | 9.33 |
| L8I442 | Inhibitor of growth protein (Fragment) | M91_11137 | 1 | 1 | 2.62% | 31370.01 | 8.2 |
| P68103 | Elongation factor 1-alpha 1 | EEF1A1 | 1 | 1 | 6.28% | 50140.28 | 9.1 |
| Q32PH8 | Elongation factor 1-alpha 2 | EEF1A2 | 1 | 1 | 6.26% | 50469.57 | 9.11 |
| L8IAH6 | Steroid receptor RNA activator 1 (Fragment) | M91_15332 | 1 | 1 | 4.68% | 25840.04 | 5.81 |
| Q3MHM7 | 60S ribosomal protein L35 | RPL35 | 1 | 1 | 8.13% | 14565.32 | 11.04 |

Table S2 The list of proteins interacting with CD18 identified by the shotgun

| Accession | Protein | Gene | Peptides | Unique Peptides | coverage | MW | PI |
| --- | --- | --- | --- | --- | --- | --- | --- |
| Q2KJH7 | Aldehyde dehydrogenase 18 family, member A1 | ALDH18A1 | 1 | 1 | 1.01% | 87193.06 | 6.86 |
| E1B7I1 | RNA-binding protein MEX3A (Fragment) | MEX3A | 1 | 1 | 1.45% | 50467.51 | 7.93 |
| P80724 | Brain acid soluble protein 1 | BASP1 | 1 | 1 | 4.85% | 23010.29 | 4.55 |
| P01576 | Interferon beta-2 | IFNB2 | 1 | 1 | 3.23% | 22318.77 | 8.6 |
| Q58DT1 | 60S ribosomal protein L7 | RPL7 | 1 | 1 | 2.42% | 29168.42 | 10.68 |
| Q32KP5 | Chromosome 20 open reading frame 71 ortholog | C13H20ORF71 | 1 | 1 | 2.10% | 27045.14 | 8.56 |
| P23004 | Cytochrome b-c1 complex subunit 2, mitochondrial | UQCRC2 | 1 | 1 | 3.53% | 48148.01 | 8.8 |
| F1N0H5 | Zinc finger CCCH domain-containing protein 13 | ZC3H13 | 1 | 1 | 0.36% | 194593.5 | 9.46 |
| L8ILV5 | Lipolysis-stimulated lipoprotein receptor (Fragment) | M91_08226 | 1 | 1 | 1.09% | 71369.93 | 8.31 |
| F1N7R5 | Coiled-coil and C2 domain-containing protein 1B | CC2D1B | 1 | 1 | 0.94% | 93400.43 | 5.2 |
| A6H750 | Kinesin-like protein KIF2B | KIF2B | 1 | 1 | 1.46% | 76906.06 | 9.3 |
| F1MTH3 | T-lymphoma invasion and metastasis-inducing protein 2 | TIAM2 | 1 | 1 | 0.36% | 185524.6 | 7.04 |
| Q0VBY4 | Tumor protein D52-like 1 | TPD52L1 | 1 | 1 | 6.70% | 22777.18 | 5.78 |
| E1BC24 | Midasin | MDN1 | 1 | 1 | 0.16% | 630687.1 | 5.41 |
| P10881 | La protein homolog | SSB | 1 | 1 | 2.97% | 46533.24 | 8.54 |
| F1MNR2 | Paired box 2 | PAX2 | 1 | 1 | 1.44% | 44705.37 | 7.25 |
| F1MC48 | Ras GTPase-activating-like protein IQGAP1 | IQGAP1 | 1 | 1 | 1.13% | 182779.8 | 6.16 |
| A7YY47 | Lamin B1 | LMNB1 | 1 | 1 | 1.71% | 66420.45 | 5.08 |
| M0QSW5 | Calcium-activated potassium channel subunit beta-3 | M91_09225 | 1 | 1 | 3.10% | 32626.3 | 9.31 |
| O18964 | Synaptojanin-1 (Fragment) | SYNJ1 | 1 | 1 | 0.45% | 146471 | 7.37 |
| P23389 | Secretogranin-1 | CHGB | 1 | 1 | 0.77% | 73338.75 | 5.19 |
| E1BF37 | MKL1/Myocardin Like 2 | MKL2 | 1 | 1 | 0.49% | 111376 | 5.5 |
| P30932 | CD9 antigen | CD9 | 1 | 1 | 3.10% | 25257.6 | 6.31 |
| F1MSM5 | Mitochondrial Poly(A) Polymerase | MTPAP | 1 | 1 | 1.20% | 65344.78 | 9.13 |

Table S2 The list of proteins interacting with CD18 identified by the shotgun

| Accession | Protein | Gene | Peptides | Unique Peptides | coverage | MW | PI |
| --- | --- | --- | --- | --- | --- | --- | --- |
| P46164 | Beta-defensin 6 | DEFB6 | 1 | 1 | 23.81% | 4838.72 | 10.74 |
| P58875 | SEC14-like protein 2 | SEC14L2 | 1 | 1 | 4.96% | 46199.83 | 8.23 |
| Q08E58 | Tubulin tyrosine ligase-like family, member 12 | TTLL12 | 1 | 1 | 1.07% | 75535.69 | 5.09 |
| Q29RM3 | Receptor expression-enhancing protein 5 | REEP5 | 1 | 1 | 6.88% | 21416.68 | 8.27 |
| Q2KI65 | Wilms tumor 1 associated protein | WTAP | 1 | 1 | 11.26% | 17767.06 | 5.42 |
| Q2KIS8 | Small nuclear ribonucleoprotein D3 polypeptide 18kDa | SNRPD3 | 1 | 1 | 7.20% | 13773.95 | 10.2 |
| Q2KJ46 | 26S proteasome non-ATPase regulatory subunit 3 | PSMD3 | 1 | 1 | 2.06% | 60955.85 | 8.65 |
| Q2T9Z7 | Kelch-like protein 9 | KLHL9 | 1 | 1 | 1.62% | 69401.84 | 5.92 |
| L8IAW9 | Kelch-like protein 13 | M91_07657 | 1 | 1 | 1.53% | 73901.21 | 6.17 |
| Q32PG1 | AP-3 complex subunit beta-1 | AP3B1 | 1 | 1 | 1.38% | 119966.5 | 5.87 |
| Q3SZP2 | Microtubule-associated protein RP/EB family member 2 | MAPRE2 | 1 | 1 | 3.99% | 36987.83 | 5.36 |
| Q3T0D9 | Ubiquitin D (Fragment) | UBD | 1 | 1 | 8.14% | 19047.03 | 9.61 |
| P30367 | Interleukin-4 | IL4 | 1 | 1 | 3.70% | 15118.7 | 9.2 |
| Q5E9P5 | Inactive serine protease PAMR1 | PAMR1 | 1 | 1 | 1.39% | 80055.93 | 7.74 |
| Q5EAE6 | Death-associated protein 1 | DAP | 1 | 1 | 8.82% | 11188.4 | 9.4 |
| Q7M3E1 | Chymotrypsin-C | CTRC | 1 | 1 | 5.60% | 29254.78 | 4.94 |
| Q2HJA9 | Phosducin-like protein | PDCL | 1 | 1 | 1.99% | 34394.27 | 4.68 |
| P02666 | Beta-casein | CSN2 | 1 | 1 | 3.13% | 25107.02 | 5.26 |
| E1BH47 | Centrosomal Protein 131 | CEP131 | 1 | 1 | 0.66% | 118851.3 | 9.14 |
| W5NV74 | Cysteinyl-TRNA Synthetase | CARS | 1 | 1 | 1.03% | 65666.78 | 6.34 |
| Q28009 | RNA-binding protein FUS | FUS | 1 | 1 | 2.73% | 52310.26 | 9.4 |
| W5P0N0 | Glutamyl Aminopeptidase | ENPEP | 1 | 1 | 0.63% | 109428.2 | 5.22 |
| W5P4G4 | RAB7B, Member RAS Oncogene Family | RAB7B | 1 | 1 | 3.00% | 22640.78 | 5.49 |
| W5P5I9 | HLF, PAR BZIP Transcription Factor | HLF | 1 | 1 | 2.71% | 33093.24 | 5.6 |
| W5P6Y6 | Adenylate Kinase 8 | AK8 | 1 | 1 | 1.88% | 54812.89 | 6.25 |
| Q3T0D0 | Heterogeneous nuclear ribonucleoprotein K | HNRNPK | 1 | 1 | 2.59% | 51018.64 | 5.14 |

| Accession | Protein | Gene | Peptides | Unique Peptides | coverage | MW | PI |
| --- | --- | --- | --- | --- | --- | --- | --- |
| F1MWB6 | SH3 Domain Containing 21 | SH3D21 | 1 | 1 | 0.74% | 74733.48 | 5.97 |
| F1N1T3 | A-kinase anchor protein 2 | AKAP2 | 1 | 1 | 1.17% | 94025.61 | 5.12 |
| Q3T165 | Prohibitin | PHB | 1 | 1 | 3.31% | 29803.71 | 5.57 |
| F1MNF0 | Limb Development Membrane Protein 1 | LMBR1 | 1 | 1 | 1.43% | 54883.02 | 5.68 |
| Q2KJH5 | Cilia- and flagella-associated protein 97 | CFAP97 | 1 | 1 | 2.28% | 58669.06 | 9.01 |
| Q3ZC00 | Lymphocyte cytosolic protein 1 (L-plastin) | LCP1 | 1 | 1 | 1.12% | 70053.31 | 5.21 |
| F1N754 | Docking Protein 7 | DOK7 | 1 | 1 | 1.09% | 47666.01 | 5.72 |
| E1BDE7 | Scaffold Attachment Factor B2 | SAFB2 | 1 | 1 | 0.52% | 108523.8 | 5.45 |
| E1BQ07 | Transmembrane protease serine | TMPRSS11A | 1 | 1 | 1.19% | 47076.54 | 9.19 |
| A6QP28 | Suv3 Like RNA Helicase | SUPV3L1 | 1 | 1 | 0.93% | 58835.12 | 6.96 |
| Q3SZC4 | NSFL1 cofactor p47 | NSFL1C | 1 | 1 | 1.35% | 40653.37 | 5.07 |
| Q2KIU7 | Radial spoke head protein 9 homolog | RSPH9 | 1 | 1 | 2.90% | 31293.56 | 5.41 |
| W5NR80 | UDP-glucuronosyltransferase | UGT2A2 | 1 | 1 | 1.54% | 53096.17 | 4.89 |

Table S2 The list of proteins interacting with CD18 identified by the shotgun
